# Protein Kinase A: A Master Kinase of Granulosa Cell Differentiation

**DOI:** 10.1038/srep28132

**Published:** 2016-06-21

**Authors:** Pawan Puri, Lynda Little-Ihrig, Uma Chandran, Nathan C. Law, Mary Hunzicker-Dunn, Anthony J. Zeleznik

**Affiliations:** 1 Department of Obstetrics, Gynecology and Reproductive Sciences, Magee Womens Research Institute, University of Pittsburgh, Pittsburgh, 15213, PA, USA; 2 Department of Biomedical Informatics, University of Pittsburgh, Pittsburgh, 15206, PA, USA; 3 School of Molecular Biosciences, College of Veterinary Medicine, Washington State University, Pullman, 99164, WA, USA.

## Abstract

Activation of protein kinase A (PKA) by follicle stimulating hormone (FSH) transduces the signal that drives differentiation of ovarian granulosa cells (GCs). An unresolved question is whether PKA is sufficient to initiate the complex program of GC responses to FSH. We compared signaling pathways and gene expression profiles of GCs stimulated with FSH or expressing PKA-CQR, a constitutively active mutant of PKA. Both FSH and PKA-CQR stimulated the phosphorylation of proteins known to be involved in GC differentiation including CREB, ß-catenin, AKT, p42/44 MAPK, GAB2, GSK-3ß, FOXO1, and YAP. In contrast, FSH stimulated the phosphorylation of p38 MAP kinase but PKA-CQR did not. Microarray analysis revealed that 85% of transcripts that were up-regulated by FSH were increased to a comparable extent by PKA-CQR and of the transcripts that were down-regulated by FSH, 76% were also down-regulated by PKA-CQR. Transcripts regulated similarly by FSH and PKA-CQR are involved in steroidogenesis and differentiation, while transcripts more robustly up-regulated by PKA-CQR are involved in ovulation. Thus, PKA, under the conditions of our experimental approach appears to function as a master upstream kinase that is sufficient to initiate the complex pattern of intracellular signaling pathway and gene expression profiles that accompany GC differentiation.

The process of granulosa cell (GC) differentiation during preovulatory follicular maturation is associated with the induction of approximately 500 target genes^1^,^2^,^3^,^4^,^5^ and is governed by the pituitary glycoprotein hormone FSH^6^. It is well known that FSH signaling in GCs is initiated by its binding to a G-protein coupled receptor (GPCR), stimulation of adenylyl cyclase, and the resulting increase in cAMP levels that activate cAMP-dependent PKA that results in phosphorylation of direct protein targets, such as CREB^7^,^8^. FSH stimulation of GCs is also associated with activation of a number of other signaling pathways including the PI3-kinase/PKB (AKT) pathway, the p42/44 MAP kinase pathway, and the p38 MAP kinase pathway that are also required for GC differentiation^9^,^10^,^11^,^12^,^13^. A major unanswered question is whether activation of PKA is sufficient to account for the complex pattern of intracellular cellular signaling that accompanies GC differentiation. To date, the only approach to investigate whether these additional signaling pathways are regulated by PKA has been through the use of PKA inhibitors such as H-89, KT 5720, and PKI. Conflicting results have been reported regarding the ability of PKA inhibitors to interfere with the ability of FSH to stimulate these additional pathways^9^,^10^,^11^,^12^,^13^. Further, because chemical inhibitors such as H-89 and KT 5720 also inhibit other intracellular kinases, often with higher affinity than towards PKA^14^, an absolute role for PKA in signaling network crosstalk in GCs cannot be definitively established. Finally, whereas studies with PKA inhibitors may indicate that PKA is necessary for the activation of signaling pathways and expression of differentiation-associated genes, inhibitors cannot reveal whether PKA alone is sufficient to do so.

Our laboratory previously reported the generation of a lentiviral vector that directs the expression of a constitutively active mutant of the catalytic subunit of PKA (PKA-CQR)^1^. This mutant does not bind effectively to the regulatory subunit of PKA and therefore does not require elevations in cAMP for stimulation of its catalytic activity^15^. PKA-CQR thus provides a unique and unequivocal tool to establish whether PKA is sufficient to account for the numerous signaling pathways that are activated by FSH in GCs as well as the program of gene expression that is essential for GC differentiation. Results of our previous study^1^ indicated that expression of PKA-CQR for 48 hr. qualitatively mimicked the stimulatory effects of FSH on the production of estradiol and progesterone by GCs as well as on the expression of the majority of genes as assessed by microarray analysis, but there were subsets of genes that were differentially regulated by FSH and PKA-CQR. However, there were two limitations with our previous study. First, we did not directly compare the effects of FSH and PKA-CQR on the activation of intracellular signaling pathways that are necessary for GC differentiation. Second, the 48 hr. stimulation window by PKA-CQR may have been sufficient to mimic the midcycle surge in luteinizing hormone (LH) and its effects on genes involved in ovulation and luteinization^16^.

In studies reported herein, we used a 24 hr. stimulation window to better reflect the initial responses of GCs to FSH and PKA-CQR. We conducted immunoblot analyses of key phospho- protein targets to directly assess the ability of PKA-CQR to activate the major signaling pathways that have previously been shown to be stimulated by FSH during the early events associated with GC differentiation. We also conducted microarray analysis to compare the gene expression profiles of GCs to identify gene networks that are regulated similarly and/or differentially by FSH and PKA during the early events of GC differentiation, prior to the potential onset of the initiation of the ovulation and luteinization program.

## Results and Discussion

### Signaling pathways activated by FSH and by PKA-CQR

The ideal experimental paradigm to establish whether PKA is sufficient to activate intracellular signaling pathways that are associated with FSH stimulation of GC differentiation would be to conduct a detailed timecourse of phospho-protein expression in response to FSH and PKA-CQR. However, the inherent difficulty in comparing the intracellular signaling pathways activated by FSH versus PKA-CQR in GCs is that the time-course for FSH activation is rapid and transient such that maximal stimulation typically occurs within the first 20 min. and declines to near basal levels by 60 min.^7^. By contrast, activation of signaling pathways by PKA-CQR is dependent upon the time required for the expression of the lentivirus-directed recombinant protein. During this period, the catalytic activity of the recombinant protein in GCs would progressively increase such that a rapid activation in PKA activity, within the time frame seen with FSH, could not be achieved. We explored a number of approaches that would enable us to acutely activate PKA-CQR in GCs. These included a chemical genetic approach of mutating the ATP binding pocket within PKA-CQR to confer unique sensitivity to a novel reversible inhibitor that could subsequently be washed out to initiate kinase activity^17^ as well as the construction of a PKA-CQR estrogen receptor fusion protein that would permit rapid activation of PKA by tamoxifen^18^. In results not reported herein, we found that neither approach was successful in generating a recombinant PKA-CQR mutant that could be acutely activated in GCs. Accordingly, we conducted a timecourse of PKA activity in GCs transduced with the lentiviral PKA-CQR using the phosphorylation of CREB (P-CREB) as an index of activity because CREB is directly phosphorylated by PKA at Ser133^8^. We found that 24 hr. post transduction with the PKA-CQR lentiviral vector was the earliest timepoint that we observed consistent phosphorylation of CREB to an extent similar to that seen following stimulation with FSH. Therefore in all experiments reported herein, we evaluated the activation of signaling pathways by PKA-CQR 24 hr. after viral transduction of GCs and compared the extent of activation of the pathways with FSH following stimulation for 20 min. and 24 hr.

#### CREB, β-catenin signaling and GSK-3β signaling

**Figure 1:**
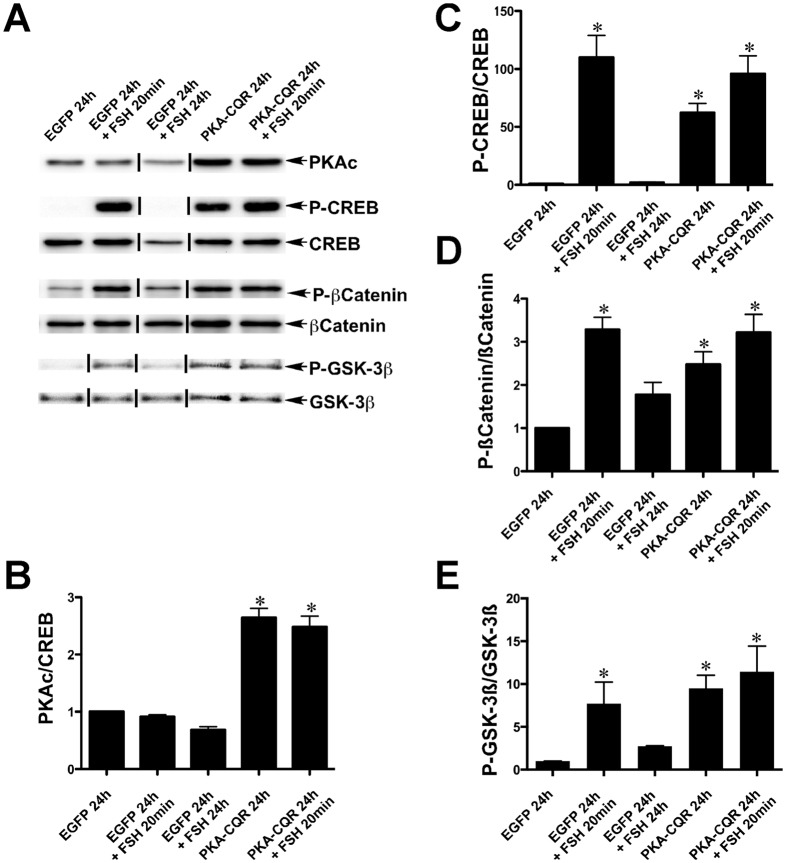
PKA-CQR expression alone is sufficient to activate CREB, β-catenin and GSK-3β signaling in undifferentiated GCs. Undifferentiated GCs were isolated, transduced with control EGFP or PKA-CQR mutant lentivirus vectors (2MOI), and stimulated with FSH as described in Methods. Whole cell protein extracts were prepared and run on SDS-PAGE gels. Separated proteins on gels were transferred and probed with antisera against PKAc, p-CREB (Ser133), CREB, p-β-catenin (Ser552), β-catenin, p-GSK-3β (Ser9) and GSK-3β as described in Methods (panel A). The densitometric quantification of the immunoblot results are shown in panels (B–E). Asterisks indicate results that are statistically different from EGFP controls (P < 0.05, n = 3). This figure contains cropped panels of immunoreactive bands representing phosphorylated and total protein levels from the same protein extracts resolved in separate immunoblots. Vertical lines between individual bands indicate bands from the same immunoblots that were rearranged for clarity of presentation. Full length blots are presented in Supplementary Fig. 1.

Immunoblot analysis demonstrated that the levels of the catalytic subunit of PKA were increased in GCs 24 hr. after transduction with the PKA-CQR lentiviral vector and elevated PKA-CQR levels were associated with increased levels of P-CREB (Fig. 1, Panels A–C, lanes 4 and 5). Stimulation by FSH for 20 min. resulted in an increase in CREB phosphorylation that declined to baseline levels by 24 hr. (Panels A and C, lanes 2 and 3). FSH stimulation of GCs for 20 min. that were previously transduced with the lentiviral PKA-CQR vector 24 hr. earlier caused a further increase in P-CREB levels indicating the continued responsiveness of CREB to FSH stimulation in cells expressing PKA-CQR (Fig. 1 Panels A,C, lane 5).

In GCs, PKA is the primary kinase that directly phosphorylates β-catenin at Ser552 to increase β-catenin-mediated transcription that is essential to up-regulate the mRNAs encoding *Lhcgr* and *Cyp19a1* that are essential for ovulation and establishment of pregnancy^19^. Both FSH stimulation (20 min.) and expression of PKA-CQR (24 hr.) increased β-catenin phosphorylation at Ser552 (Fig. 1, Panels A,D, lanes 2, 4 and 5). Another well-known direct target of PKA is GSK-3β, a potential regulator of apoptosis and steroidogenesis in GCs^20^. Both FSH stimulation and PKA-CQR expression increased GSK-3ß phosphorylation at Ser9 (Fig. 1, Panels A,E, lanes 2, 4 and 5). Taken together, these data show that PKA-CQR is sufficient to mimic the effects of FSH on the stimulation of CREB, β-catenin, and GSK-3β signaling in GCs.

#### Akt Signaling

**Figure 2:**
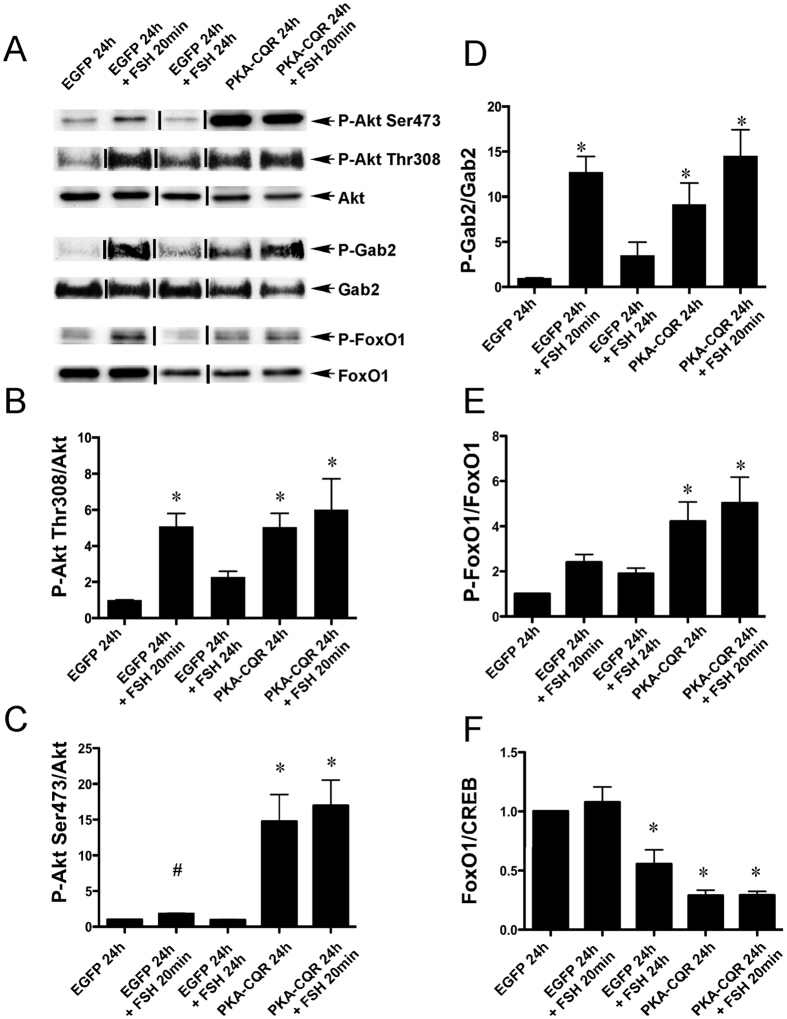
PKA-CQR expression alone is sufficient for the activation of Akt signaling in undifferentiated GCs. Primary GC cultures were established from 24-day-old female rats, transduced with control EGFP or PKA-CQR mutant lentivirus vectors (2MOI), and stimulated with FSH as described in Methods. Whole cell protein extracts, prepared from these cells were analyzed by immuno-blotting with following antisera that recognize proteins involved in the Akt signaling pathway: p-Akt (Ser473), p-Akt (Thr308), Akt, p-Gab2 (Ser159), Gab2, p-FOXO1 (Ser256) and FOXO1 (panel A). The densitometric quantification of the immunoblot results are shown in panels (B–F). Asterisks indicate results that are statistically different from EGFP controls (P < 0.05, n = 3) and # indicates that Akt Phosphorylation levels in EGFP 24 hr.+ FSH 20 min. is statistically different from EGFP 24 hr. by Student’s t-test. This figure contains cropped panels of immunoreactive bands representing phosphorylated and total protein levels from the same protein extracts resolved in separate immunoblots. Vertical lines between individual bands indicate bands from the same immunoblots that were rearranged for clarity of presentation. Full length blots are presented in Supplementary Fig. 2.

Activation of the phosphatidylinositol-3 (PI3K)/Akt pathway by FSH is believed to be PKA dependent, based on the ability of the PKA inhibitor PKI to block Akt phosphorylation on both Thr308 and Ser473^13^. The PI3K/Akt pathway is necessary for the induction by FSH of *Ccnd2*, *Cyp19a1*, *Cyp11a1*, *Lhcgr*, and other critical genes that mark the fully differentiated preovulatory GC^13^,^21^,^22^,^23^. FSH also promotes, in a PKA-dependent manner, the phosphorylation of both GAB2 on Ser159 and IRS-1 on Tyr989, phosphorylation events which are believed to facilitate recruitment and activation of PI3K to IRS-1^9^. Both PKA-CQR and FSH increased Akt phosphorylation on Thr308 and Ser473 residues (Fig. 2, Panels A–C, lanes 2, 4 and 5) and GAB2 phosphorylation on Ser159 (Fig. 2, Panels A,D, lanes 2, 4 and 5). Student-T test was used to compare vehicle versus FSH for Ser473 because the lack of significance using analysis of variance related to the larger and more variable signal seen with PKA-CQR. These results indicate that PKA-CQR is sufficient to activate the PI3K/Akt pathway in GCs. In further support of this conclusion, FOXO1, a transcription factor known to be phosphorylated by Akt on Ser256, was also phosphorylated in response to PKA-CQR and FSH (Fig. 2, Panels A,E, lanes 2, 4 and 5). Further, total protein levels of FOXO1 were reduced in GCs which expressed constitutively active PKA-CQR or were stimulated by FSH for 24 hr. (Fig. 2, Panels A,F, lanes 3, 4 and 5). This decrease in total FOXO1 levels is consistent with the known degradation of FOXO1 after its phosphorylation by Akt^24^. Collectively, these findings demonstrate that PKA-CQR is sufficient to activate the Akt signaling pathway in GCs.

#### p42/44 MAPK and Src signaling

**Figure 3:**
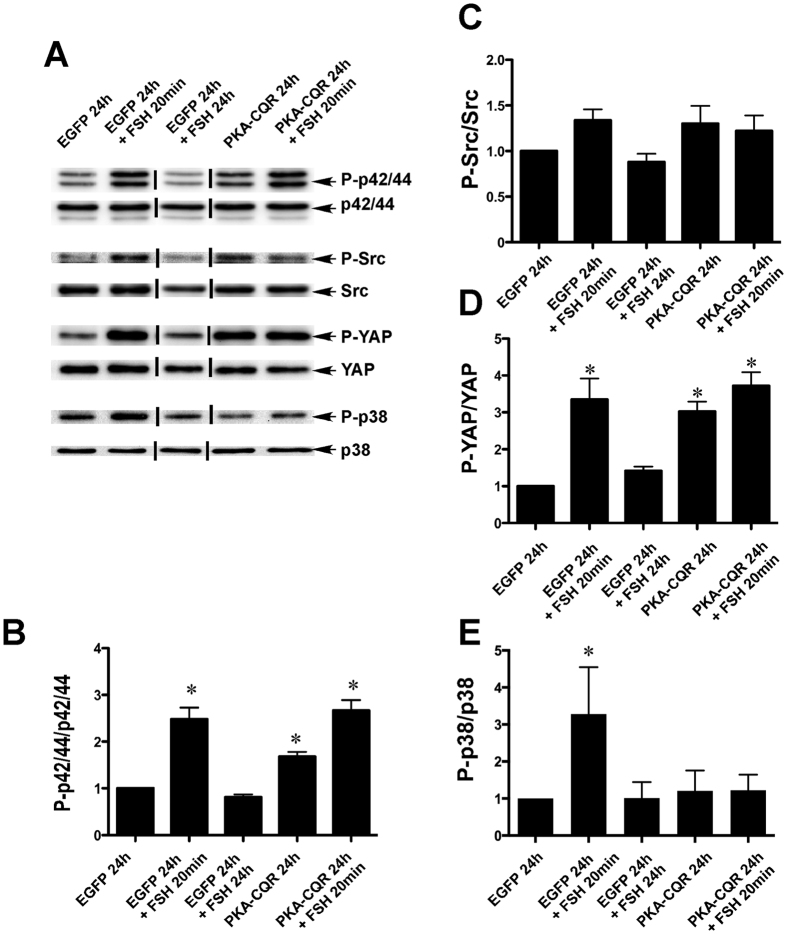
PKA-CQR expression alone is sufficient to activate p42/44 MAPK and Hippo signaling pathways but does not stimulate p38 MAPK signaling. Primary GC cultures were established from 24 day-old female rats, transduced with control EGFP or PKA-CQR mutant lentivirus vectors (2MOI), and stimulated with FSH as described in Methods. Whole cell protein extracts were prepared from all the control and treatment groups and run on SDS-PAGE gels. Separated proteins on gels were transferred and probed with indicated antisera (panel A).The densitometric quantification of the immunoblot results are shown in panels (B–F). Asterisks indicate results that are statistically different from EGFP controls (P < 0.05, n = 3). This figure contains cropped panels of immunoreactive bands representing phosphorylated and total protein levels from the same protein extracts resolved in separate immunoblots. Vertical lines between individual bands indicate bands from the same immunoblots that were rearranged for clarity of presentation. Full length blots are presented in Supplementary Fig. 3.

FSH stimulation of GCs results in a transient increase of p42/44 MAPK phosphorylation^10^,^25^. The p42/44MAPK (MAPK1/3) pathway is required for the production of estradiol by way of its stimulation of the differentiation-associated transcripts *Cyp19 Egfr, Inha, Egr1*, and *Ereg*^25^,^26^,^27^. The PKA dependence of p42/44 MAPK phosphorylation is controversial because PKA inhibitors have been shown to either block^25^,^26^,^27^ or have no effect^10^ on the FSH-mediated phosphorylation of this kinase. In studies that indicate a role for PKA in p42/44 MAPK activation, stimulation by PKA is thought to be indirect and involves phosphorylation/inactivation of a protein phosphatase resulting in its dissociation from p42/44 MAPK and leading to activation of the signaling pathway^25^. Our results (Fig. 3, Panels A,B, lanes 2, 4 and 5) confirm that FSH is able to stimulate this pathway and show further that constitutively activated PKA alone is sufficient to cause a robust increase in p42/44 MAPK phosphorylation. An additional question in relation to FSH-mediated p42/44 MAPK activation is the requirement for stimulation of Src activity, as the Src-inhibitor PP2 can block FSH-mediated increases in RAS^10^ and p42/44 MAPK activation^10^,^25^. Our results show that neither FSH stimulation nor constitutively active PKA significantly increased phosphorylation of Src at Tyr416 (Fig. 3, Panels A,C). These results are consistent with the previous findings that FSH does not activate Src but that Src is required in GCs for FSH-mediated activation of the p42/44 MAPK signaling^25^.

#### Hippo signaling

The highly conserved Hippo signaling pathway is involved in cell proliferation, cell death and differentiation. This pathway is activated by a series of kinases that ultimately promote the phosphorylation and cytoplasmic retention of Yes associated protein (YAP), thereby abrogating its promotion of gene transcription and curtailing cell proliferation^28^. In humans suffering from premature ovarian failure, ovarian fragmentation results in YAP dephosphorylation, increased production of CCN (also known as CTGF) growth factors and the stimulation of the growth of small preantral follicles^29^,^30^.

As shown in Fig. 3 (Panels A,D, lanes 2, 4 and 5), both FSH stimulation and PKA-CQR expression in GCs increased phosphorylation of YAP at Ser 127, the position known to promote its nuclear exclusion^28^. This observation is consistent with recent studies demonstrating that direct ligand stimulation of Gαs, cAMP analogs, or transfection of the catalytic subunit of PKA results in YAP phosphorylation in a number of cell types^31^,^32^ . This effect of PKA appears to be due to the phosphorylation and inhibition of RhoA which, in turn, relieves inhibition of Lats kinase leading to phosphorylation of YAP^32^.

Although both FSH and constitutively active PKA stimulated the phosphorylation of YAP, this effect at first glance would not be consistent with the known mechanisms of follicular growth because phosphorylation of YAP would be expected to suppress proliferation^28^ whereas FSH promotes follicular development by enhancing GC proliferation and inhibiting apoptosis^33^. It is possible that the phosphorylation and nuclear exclusion of YAP by FSH and PKA-CQR in GCs may contribute to the stimulation of other cellular processes associated with ovarian function such as differentiation. In this regard, it has been noted that forskolin-induced differentiation of murine 3T3-L1 cells into adipocytes is associated with increased phosphorylation of YAP that is repressed by the PKA inhibitor KT5720 and mimicked by knockdown of YAP^32^. Alternatively, as recent evidence suggests that the loss of Hippo signaling in Drosophila enhances the PI3-kinase/AKT pathway^34^, it is possible that the phosphorylation of YAP may also contribute to the ability of FSH and PKA-CQR to activate the PI3-kinase/AKT pathway in GCs.

#### p38 MAPK signaling

Phosphorylation of p38 MAPK (MAPK14) is stimulated by FSH and thought to be dependent on PKA, based on the ability of the PKA inhibitor H-89 to abrogate this response^11^. FSH-stimulated phosphorylation of p38 MAPK has been linked with GC rounding and regulation of estrogen and progesterone production^11^,^12^. We confirmed that stimulation of GCs with FSH for 20 min. increased the levels of phosphorylated p38 MAPK (Thr180/Tyr182; Fig. 3, Panels A,E, lane 2). However, expression of PKA-CQR did not result in increased p38 MAPK phosphorylation (lane 4). Thus, constitutively active PKA alone under these experimental conditions was not sufficient to activate this pathway. In addition, p38 MAPK became unresponsive to FSH in GCs expressing PKA-CQR as phosphorylation of this kinase was not increased following 20 min. of stimulation by FSH in cells previously transduced with PKA-CQR (Fig. 3, Panels A,E, lane 5). This observation is consistent with a previous report^35^ that inhibition of PKA by H-89 augmented FSH-stimulated phosphorylation of p38 MAPK. Thus, whereas p38 MAPK is rapidly activated by FSH, it remains unclear as to whether this activation is PKA-dependent. Moreover, prolonged stimulation by PKA renders this kinase unresponsive to FSH by a yet to be identified mechanism.

### Comparison of gene expression profiles of GCs stimulated with FSH or expressing constitutively active PKA

**Figure 4:**
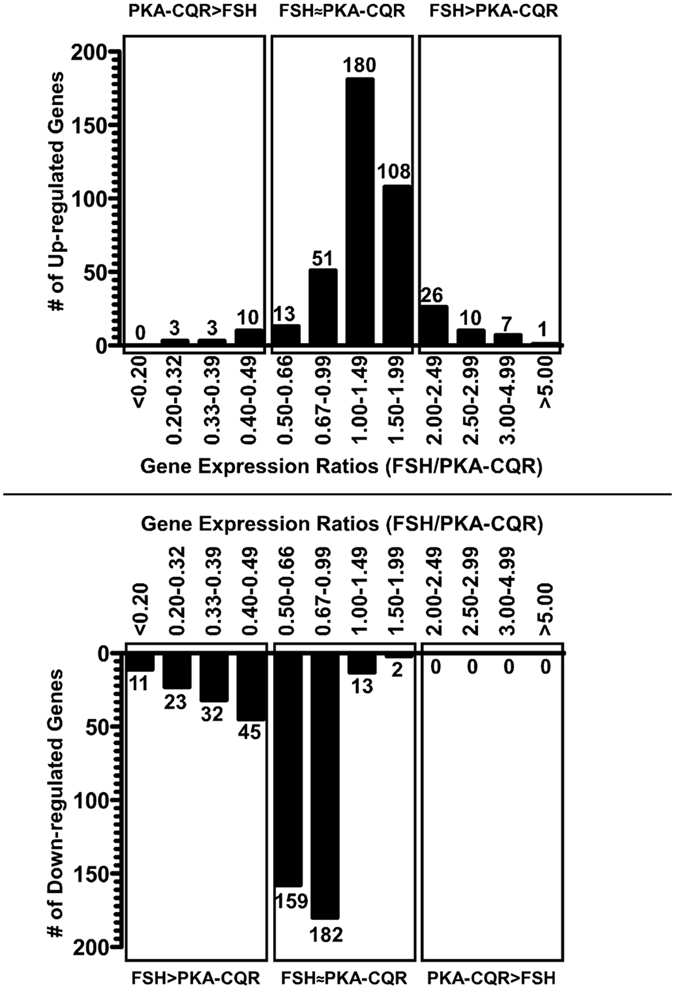
Comparison of gene expression profiles at 24 hr. of GCs stimulated with FSH or expressing PKA-CQR by relative gene expression ratio metric. Transcripts up-regulated (**A**) or down-regulated (**B**) at least 2-fold by FSH or PKA-CQR were identified by dividing the expression values of individual transcripts in the FSH or PKA-CQR groups (n = 3) with the corresponding expression values of the respective transcript levels in the control groups (n = 2). To determine the up-regulated and down-regulated transcripts for the third group, the average of control values of group 1 and group 2 were used. For transcripts altered 2-fold or more by either FSH or PKA-CQR, relative expression change ratios were calculated by dividing the fold-change of each transcript in the FSH group by the fold change of that transcript in the PKA-CQR group. The ratios were categorized into the indicated ranges and the number of up-regulated (**A**) or down-regulated genes (**B**) that fell into each range was determined. Transcripts with less than 2-fold difference between FSH and PKA-CQR were considered similarly regulated by FSH and PKA-CQR (FSH~PKA).

We compared mRNA expression levels of approximately 14,000 transcripts in GCs stimulated with FSH or expressing PKA-CQR to determine whether PKA activation alone mimics the FSH-mediated gene expression profiles during the initial 24 hr. of stimulation. We developed the relative expression change ratio metric, which divided the fold-change following stimulation with FSH by the fold change following stimulation by PKA-CQR for any gene that was altered 2-fold or more by either FSH or PKA-CQR. Ratios that were 0.5 to 1.99 represented genes that were regulated similarly by FSH and PKA-CQR (less than 2-fold differences between the two stimulators) (FSH~PKA-CQR); ratios of less than 0.5 and more than 1.99 represented transcripts that were regulated more than 2-fold by PKA-CQR (PKA-CQR > FSH) or by FSH (FSH > PKA-CQR), respectively (Fig. 4A,B). A complete list of transcripts whose expression levels changed by more than two fold (increase or decrease) is presented in Supplementary Table S1.

#### Up-regulated genes

We found that 85% (352 genes) fell under the FSH~PKA-CQR category (Fig. 4A, Center Panel and S1). Eleven % (44 genes) had fold-change ratios of more than 2.0 and were thus stimulated at least 2-fold more by FSH than PKA-CQR (Fig. 4A, Right Panel and Supplementary Table S1). Of these, only 7 were up-regulated 3-fold greater by FSH and only one gene was stimulated more than 5-fold more by FSH (Fig. 4A, Right Panel and Supplementary Table S1). Further analysis of the group of 44 transcripts that were increased more than two-fold by FSH did not reveal any common upstream regulators that might account for their preferential up-regulation by FSH.

Four % (16 genes) had a fold-change ratio less than 0.5, indicating that PKA-CQR was at least 2-fold more effective than FSH in stimulating their expression (Fig. 4A, Left Panel and S1). Only three genes were up-regulated by PKA-CQR by more than 3-fold; the maximum fold increase was 3.87 (Supplementary Table S1). As described below, the differential activation P38 MAPK by FSH and PKA-CQR may account for the differential regulation of this group of transcripts.

#### Down-regulated genes

FSH stimulation or PKA-CQR expression resulted in at least a 2-fold down-regulation of 461 genes and 168 genes, respectively. The relative expression ratios of 76% (356 genes) of these down-regulated genes fell between 0.5–1.99. Thus, PKA-CQR partially mimics FSH-mediated down-regulation of GC gene expression (Fig. 4B, Central Panel and Supplementary Table S1).

FSH decreased the expression of 24% (111 transcripts) by 2-fold or more compared to PKA-CQR (Fig. 4B, Left Panel and Supplementary Table S1). In contrast to the similar abilities of FSH versus PKA-CQR to up-regulate genes in GCs, under the conditions of our experiment FSH appears to be more effective than PKA-CQR in down-regulating gene expression. Specifically, FSH was 3-fold or 5-fold more effective in the down-regulation of 23 and 11 transcripts, respectively. PKA-CQR did not down-regulate any gene 2-fold more effectively than FSH (Fig. 4B, Right Panel and Supplementary Table S1). These results suggest that FSH-mediated down-regulation of GC genes may involve other mechanisms in addition to the activation of PKA. However, further analysis of the group of 111 transcripts that were decreased more than two-fold by FSH than PKA-CQR did not reveal any common upstream regulators that might account for their preferential down-regulation by FSH.

### Functional annotation of transcripts regulated similarly or differentially by FSH and PKA-CQR identified by DAVID analysis

Supplementary Tables S2–S4 present functional annotations of transcripts that were regulated to a similar extent (fold-change ratio less than 2) by FSH and PKA-CQR, transcripts that were regulated to a greater extent (fold-difference greater than 2) by FSH than by PKA-CQR and transcripts that were regulated to a greater extent (fold-difference greater than 2) by PKA-CQR than by FSH. Of these as well as those presented in Supplementary Table 1, we selected transcripts that have been shown to be associated with major functions in GCs as described below.

#### Transcripts associated with steroidogenesis and GC differentiation

Stimulation by FSH or PKA-CQR for 24 hr. comparably induced the expression of transcripts associated with GC steroidogenesis and differentiation including the cholesterol transporter protein, *Star,* as well as *Cyp19a1 and Cyp11a1*, the rate limiting enzymes for estrogen and progesterone synthesis, respectively (Table 1). Both PKA-CQR and FSH treatment also stimulated markers of GC differentiation: luteinizing hormone receptor (*Lhcgr*); epidermal growth factor receptor (*Egfr*) and inhibin subunits *Inha, Inhba* and *Inhb* (Table 1). The relative expression ratios for all these genes except *Cyp19a1* were between 0.5–2.0 indicating comparable regulation by PKA-CQR and FSH.

The expression of many of the GC differentiation markers and steroidogenic genes is under the control of the transcription regulators *Nr5a1*, *Nr5a2*, *Gata4 and Foxo1*^36^,^37^,^38^,^39^. Microarray analysis showed that mRNAs encoding these transcription factors were increased to a similar extent after FSH stimulation or PKA-CQR expression (Table 1). FSH is also known to down-regulate the expression of *Foxo1* and *Dax-1* (*Nr0b1*) to relieve their inhibitory effects on the expression of *Cyp19A, Cyp11A1*, *Lhcgr* and the transcription factors *Nr5a1* and *Nr5a2*^2^,^19^,^21^. Both PKA-CQR and FSH down-regulated mRNA levels of *Foxo1* to a similar extent (Table 1), consistent with the decline in FOXO1 protein levels (see Fig. 2). Although both FSH and PKA-CQR down-regulated *Nr0b1*, its expression was reduced to a greater extent by FSH than by PKA-CQR.

It is well-established that CREB, β-catenin, Akt, and p42/44 MAPK are major upstream regulators of the transcriptional machinery that modulates mRNA levels of genes associated with cholesterol and steroid biosynthesis, differentiation markers such as *Lhcgr* and *Inha*, and proliferation in GCs^19^,^27^,^40^,^41^,^42^,^43^. Our results showed that PKA-CQR mimics FSH in activating all of the above-mentioned signaling cascades. Thus, the abilities of FSH and PKA-CQR to augment the expression of mRNA markers of steroidogenesis and differentiation are consistent with their similar activation of the aforementioned signaling pathways.

#### Genes involved in ovulation and luteinization

Whereas the responses to both FSH and LH are mediated primarily by cAMP/PKA, these two hormones appear to elicit distinct effects on the gene expression profiles of preantral (immature) GCs and preovulatory (mature) GCs, respectively. For example, FSH stimulated follicular development is associated with the up-regulation mRNAs encoding *Cyp19a1* and *Lhcgr* but not ovulation-associated genes such as *Pgr* and *Ptgs2*^6^,^7^. In contrast, LH stimulated ovulation is associated with the up-regulation of mRNA levels for *Pgr* and *Ptgs2* and the down-regulation of mRNA levels for *Cyp19a1* and *Lhcgr*^6^,^7^. These differences in the FSH and LH responses have been attributed to the distinct proteomes present at the two different stages of GC maturation, the efficiency of coupling of the FSH and LH receptors to distinct pathways, and/or differences in the extent or duration of cAMP/PKA signaling between FSH and LH^6^,^7^. In our current study, although FSH stimulation of preantral GCs showed an increase in the expression of ovulation-associated genes *Areg, Ereg, Pgr* and *Ptgs2,* expression of PKA-CQR resulted in higher expression of these ovulation-associated genes than FSH (Table 1).

The preferential increase in mRNAs for proteins involved in ovulation by PKA-CQR as compared to FSH may be due to the differential effects of FSH and PKA-CQR in the activation of p38 MAPK. Previous work by others demonstrated that FSH stimulation of GCs obtained from p38 MAPK-null mice resulted in higher mRNA levels of the ovulation-associated genes *Areg, Ereg, Pgr*, and *Tnfaip6* compared to GCs from wild type animals, indicating that p38 MAPK activation has a negative regulatory effect on the expression of these ovulation-associated genes^44^. In our current study, p38 MAPK was the only kinase cascade that was not activated by PKA-CQR. In fact, we observed that p38 MAPK in GCs expressing PKA-CQR for 24 hr. became unresponsive to acute (20 min.) FSH stimulation. These data tempt the speculation that activated p38 MAPK in immature GCs may prevent the premature expression of the ovulation associated genes such as *Areg, Ereg, Pgr* and *Tnfaip6* and that elevated expression of these genes at the time of ovulation may be due to a cAMP/PKA-mediated reduction in p38 MAPK activity.

Comparison of gene expression patterns conducted at 24 hr. with our previous microarray study conducted at 48 hr. revealed that many genes such as *P450scc*, *Star* and *Inhba* were regulated comparably by FSH and PKA-CQR at both time-points^1^. Ovulation-associated transcripts such as *Areg, Ereg* and *Ptgs2* were up-regulated to a greater extent by PKA-CQR than FSH at both time-points. However, at the 24 hr. time-point, PKA-CQR more robustly up-regulated *Cyp19a1* (59.7-fold) than FSH (29.4-fold) but at the 48 hr. time-point, FSH more robustly up-regulated *Cyp19a1* than did PKA-CQR (260-fold vs. 58-fold respectively). A similar pattern was also observed for the *Lhcgr* expression. The differential regulation of *Cyp19a1* and *Lhcgr* transcripts by FSH and PKA-CQR at these two time-points could be due to the progression of the luteinization program to an advanced stage by 48 hr. in PKA-CQR expressing GCs as the expression of *Areg* and *Ereg* results in termination of the follicular differentiation program and initiation of luteinization that is associated with down-regulation of *Lhcgr* and *Cyp19a1*^45^,^46^. As noted above, the differential actions of FSH and PKA-CQR on the phosphorylation of p38MAPK could be responsible for the preferential stimulation of ovulation-associated genes and initiation of the luteinization program. In this regard, pharmacological inhibition of p38MAPK has been shown to reduce FSH-stimulated estrogen production while augmenting FSH-stimulated progesterone production, both of which occur during luteinization of GCs^12^.

A notable difference between stimulation of GCs by FSH and PKA-CQR is that the mutations in PKA-CQR render the catalytic subunits unable to bind effectively to the regulatory subunits that serve as docking sites for A-kinase anchoring proteins (AKAPs)^15^. AKAPs target the native PKA holoenzyme to specific cellular microdomains that include substrates, phosphodiesterases and phosphatases, all of which serve to provide discreet spatiotemporal control of cellular functions^47^,^48^,^49^. Whereas FSH-stimulated activation of PKA that is tethered to AKAPs would be expected to generate a specific pattern of phosphorylation events, PKA-CQR, because it does not tether to AKAPs, would be expected to lead to the indiscriminant phosphorylation of PKA substrates within the cell. In addition, the temporal pattern of phosphorylation events triggered by FSH in our study also differs from that of the constitutively active PKA-CQR in that the FSH stimulus was rapid and transient while phosphorylation in response to PKA-CQR occurred over a period of 24 hr. (Figs 1, 2, 3). Despite these differences in the spatiotemporal signaling patterns between FSH and PKA-CQR, we find it remarkable that we observed such striking similarities in activation of signaling pathways and gene expression profiles between the two different stimuli. However, it is possible that AKAPs may fine-tune the FSH-stimulated differentiation program that could account for the minor differences in the phosphorylation patterns and gene expression profiles in GCs that were stimulated by FSH or PKA-CQR.

In conclusion, taking into consideration the aforementioned caveats in our approach, our results are consistent with the hypothesis that PKA is a proximal kinase downstream of the FSH receptor that not only directly regulates classical targets such as CREB and nonclassical targets such as ß-catenin, but also regulates intracellular signaling cascades that are traditionally activated by growth factors via their receptor tyrosine kinases, such as the PI3K/AKT and the p42/p44 MAPK signaling pathways, to direct a program of GC function that is essential for female fertility^7^. Because PKA is ubiquitously expressed in all cells and functions to regulate a multitude of cellular responses, our unprecedented findings in GCs that it functions as an upstream master kinase for both classical Ser/Thr targets as well as targets of receptor tyrosine kinases indicates that this spectrum of activities could be a more universal response to agonists that signal via cAMP and PKA than previously appreciated. GCs thus offer a physiologically relevant *in vitro* cell model to determine the mechanisms by which a single protein kinase (PKA) can branch out to direct multiple signaling networks that control cellular functions.

## Methods

### Chemicals and Reagents

Human FSH (AFP-4161-B; 3205 IU Second International Reference Preparation of FSH/mg) was provided by the National Hormone and Pituitary Program (National Institute of Diabetes and Digestive and Kidney Diseases, National Institutes of Health, Bethesda, MD). The generation and production of the PKA-CQR lentivirus has been described previously^1^. The lentivirus directing the expression of EGFP was obtained from the Magee-Womens Research Institute Transgenic and Molecular Research Core (Pittsburgh, PA).

### Granulosa Cell Culture and Lentiviral Transduction

All experimental protocols involving the use of animal were approved by and conducted in accordance with the guidelines of the University of Pittsburgh Institutional Animal Care and Use Committee. Sexually immature female rats (24 day old) were purchased from Hilltop Lab Animals (Scottdale, PA) and ovaries were isolated and GCs collected as described previously^1^. For viral transduction, isolated granulosa cells were suspended into M199 containing 8 μg/ml polybrene (hexadimethrin bromide, catalog item 52495; Fluka/Sigma-Aldrich Corp., St. Louis, MO). For each well of a 12-well culture plate, GCs were placed into individual 5 ml polypropylene culture tubes in a total volume of 0.3 ml that contained 8 μg/ml polybrene, ~2.5 × 10^6^ GCs and 5 × 10^6^ PFU of either the PKA-CQR or EGFP lentiviral vectors. Tubes were centrifuged in an Eppendorf 5810 R centrifuge for four 30 min. intervals at 1,200 × g at 37 C. After each 30 min. centrifugation, cell pellets were gently resuspended and after the final centrifugation, the entire 0.3 ml of the transduction mixture was transferred into individual wells of a 12-well tissue culture plates previously coated with donor calf serum that contained 0.7 ml M199 with 30 ng/ml testosterone. Cells were exposed to FSH at the time of plating as described in the figure legends.

### Western Immunoblot Analysis

GC monolayers were washed with PBS and collected in approximately 150 μl 1 × sodium dodecyl sulfate-sample buffer (62.5 mM Tris-HCl, pH 6.8, 2% wt/vol sodium dodecyl sulfate, 10% glycerol, 50 mM dithiothreitol, 0.01% wt/vol pyronin Y). Whole-cell extracts were sonicated in an ice bath with a sonic dismembrator (Model 100; Fisher Scientific Co. LLC, Pittsburgh, PA) for 10 sec at a power setting of 5 and boiled 5 min. Immunoblots for P-GAB2(Ser159), P-GSK3ß(Ser9), P-AKT(Thr308), total GSK3, total p38 MAPK, and total GAB2 were performed at Washington State University as previously described^2^,^11^,^13^ using chemiluminescent detection with X-ray film. Resultant images were digitized and quantified using ImageJ software (National Institutes of Health, Bethesda, MD). Immunoblots for the remaining phosphorylated and total protein levels were conducted at the University of Pittsburgh. For these samples, whole-cell extracts were separated on a 12% SDS-PAGE and resolved proteins were transferred to nitrocellulose membranes. Chemiluminescent detection was accomplished using the BM chemiluminescence Western blotting kit (Roche Diagnostics Corporation, Indianapolis, IN) with anti-rabbit or anti-mouse horseradish peroxidase-conjugated secondary antibody (Sigma, St. Louis, MO) diluted according to manufacturer’s directions. Digital images were captured using the FluorChem E digital imager (ProteinSimple, San Jose, CA). Densitometric analysis of digitized images was performed using the program Alphaview SA, version 3.4.0.0 (ProteinSimple, San Jose, CA). The FluorChem E digital capture system is programmed to capture non-saturated images and the AlphaView software associated with it utilizes digital images captured in the linear range to calculate densitometries of bands. Supplementary Table S5 lists the antibodies and blocking conditions for all immunoblots.

### DNA Microarray Study

Three separate groups of GCs were collected and placed into primary culture as described above and subjected to the following treatments: (1) EGFP lentivirus (2 MOI) plus testosterone (30 ng/ml), (2) EGFP lentivirus (2 MOI) plus FSH (100 ng/ml) and testosterone (30 ng/ml), (3) PKA-CQR lentivirus (2 MOI) plus testosterone (30 ng/ml). Total RNA was extracted from the cells 24 hr. later using RNA-Bee solution, frozen at −80 C, and transported to the Genomics and Proteomics Core Laboratory of the University of Pittsburgh to perform microarray analysis with the Affymetrix Rat Genome 230 2.0 array as per the manufacturer’s instructions. For data analysis, MAS5.0 normalized Affymetrix gene expression data were imported into BRB Array Tools (http://linus.nci.nih.gov/BRB-ArrayTools.html) for differential expression (DE) analysis. DE genes were identified using the Student t test (p < 0.001) in BRB. The CEL files and the processed normalized data have been submitted to Gene Expression Omnibus (GEO) under series record GSE73955.

### Database for Annotation Visualization and Integrated Discovery (DAVID) Analysis

Differentially expressed genes were then uploaded and analyzed by DAVID bioinformatics resource (https://david.ncifcrf.gov/tools.jsp). We performed analyses on three different groups of genes. (1) genes that had expression ratio between 0.5–2.0 i.e. were up and down regulated to a similar extent by FSH and PKA activation. (2) genes that were up-regulated and downregulated 2-fold or more by FSH than PKA. (3) genes that were up-regulated 2-fold or more by PKA than FSH.

### Statistics

The mean ± SEM of relative signal intensities were determined from at least three independent experiments. All quantitative comparisons between treatment groups were conducted with protein extracts analyzed on the same immunoblot. Results were analyzed by ANOVA and Newman-Keuls or Student’s t-test at a 5% significance level utilizing GraphPad Prism 4.3 (GraphPad Software, San Diego, CA).

## Additional Information

**How to cite this article**: Puri, P. *et al.* Protein Kinase A: A Master Kinase of Granulosa Cell Differentiation. *Sci. Rep.* **6**, 28132; doi: 10.1038/srep28132 (2016).

## Supplementary Material

Supplementary Information

## Figures and Tables

**Table 1:** Effects of FSH (24 hr.) or PKA-CQR (24 hr.) on transcripts associated with GC steroidogenesis, differentiation and ovulation.

Symbol	Entrez Gene Name	FSH/Con	PKA/Con	FSH/PKA
Genes involved in steroidogenesis				
* Srebf1*	sterol regulatory element binding transcription factor 1	2.28	1.8	1.26
* Star*	steroidogenic acute regulatory protein	14.13	9.11	1.55
* Cyp19a1*	cytochrome P450, family 19, subfamily A, polypeptide 1	29.44	59.71	0.49
* Cyp11a1*	cytochrome P450, family 11, subfamily A, polypeptide 1	51.31	36.4	1.41
* Por*	P450 (cytochrome) oxidoreductase	2.3	1.95	1.18
Genes involved in differentiation				
* Egfr*	epidermal growth factor receptor	2.03	2.04	1
* Lhcgr*	luteinizing hormone/choriogonadotropin receptor	27.99	41.79	0.67
* Prlr*	prolactin receptor	5.44	4.08	1.33
* Ctgf*	connective tissue growth factor	0.59	0.82	0.73
* Inha*	inhibin, alpha	2.43	2.41	1.01
* Inhba*	inhibin, beta A	2.77	2.5	1.11
* Inhbb*	inhibin, beta B	3.43	3.35	1.02
Transcriptional regulators involved in GC differentiation				
* Nr0b2*	nuclear receptor subfamily 0, group B, member 2	2.77	1.67	1.66
* Nr5a1*	nuclear receptor subfamily 5, group A, member 1	2.36	2.05	1.15
* Nr5a2*	nuclear receptor subfamily 5, group A, member 2	2.35	1.88	1.25
* Nr1h3*	nuclear receptor subfamily 1, group H, member 3	2.44	1.98	1.23
* Cebpb*	CCAAT/enhancer binding protein (C/EBP), beta	2.02	1.77	1.14
* Giot1*	Zfp932	5.71	6.07	0.94
* Gata4*	GATA binding protein 4	2.31	2.25	1.02
* Nr0b1*	nuclear receptor subfamily 0, group B, member 1	−5.93	−1.83	3.24
* Foxo1*	forkhead box O1	−2.13	−1.41	1.51
* Tead3*	TEA domain family member 3	−2.06	−1.75	1.18
Genes involved in ovulation				
* Areg*	amphiregulin	3.57	13.84	0.26
* Ereg*	epiregulin	5.37	18.1	0.3
* Pgr*	progesterone receptor	1.74	4.29	0.41
* Snap25*	synaptosomal-associated protein 25Kda	1.6	2.57	0.62
* Tnfaip6*	tumor necrosis factor, alpha-induced protein 6	30.15	46.64	0.65
* Ptgs2*	prostaglandin-endoperoxide synthase 2	12.77	35.2	0.36
