# Supplementary material for: Protein Kinase A: A Master Kinase of Granulosa Cell Differentiation
Source: Sci Rep. 2016 Jun 21;6:28132. doi: 10.1038/srep28132 (PMC4914995; doi:10.1038/srep28132)
Supplement: Supplementary Information [file srep28132-s1.pdf]

Protein Kinase A: A Master Kinase of Granulosa Cell Differentiation

Pawan Puri[a], Lynda Little-Ihrig[a], Uma Chandran[b], Nathan C Law[c], Mary Hunzicker-Dunn[c], Anthony J. Zeleznik[a*]

[a]Department of Obstetrics, Gynecology and Reproductive Sciences, Magee Womens Research Institute, University of Pittsburgh, Pittsburgh, PA, 15213 [b]Department of Biomedical Informatics, University of Pittsburgh, Pittsburgh, PA, 15206 [c]School of Molecular Biosciences, College of Veterinary Medicine, Washington State University, Pullman, WA, 99164

***Corresponding author:**

Anthony J. Zeleznik, Ph.D.

Department of Obstetrics Gynecology and Reproductive Sciences

Magee Womens Research Institute

204 Craft Ave., Room B309

Pittsburgh, PA 15213

412-641-7673

zeleznikaj@mwri.magee.edu

Supplementary Figure 1. Uncropped blots used in the construction of composite of Figure 1 in text. Numbers in parentheses correspond to lane identification numbers in Figure 1.

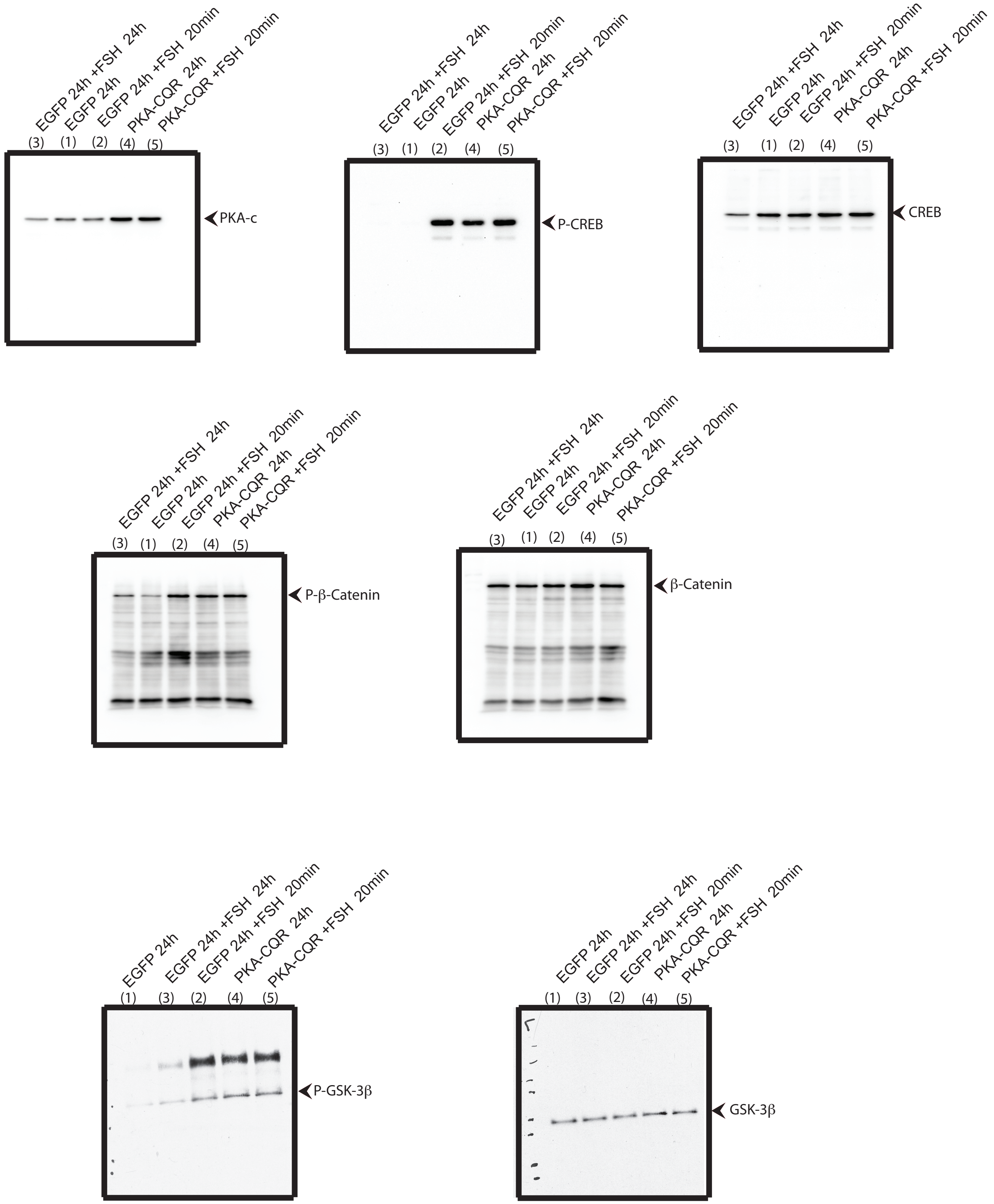

Supplementary Figure 2. Uncropped blots used in the construction of composite of Figure 2 in text. Numbers in parentheses correspond to lane identification numbers in Figure 2.

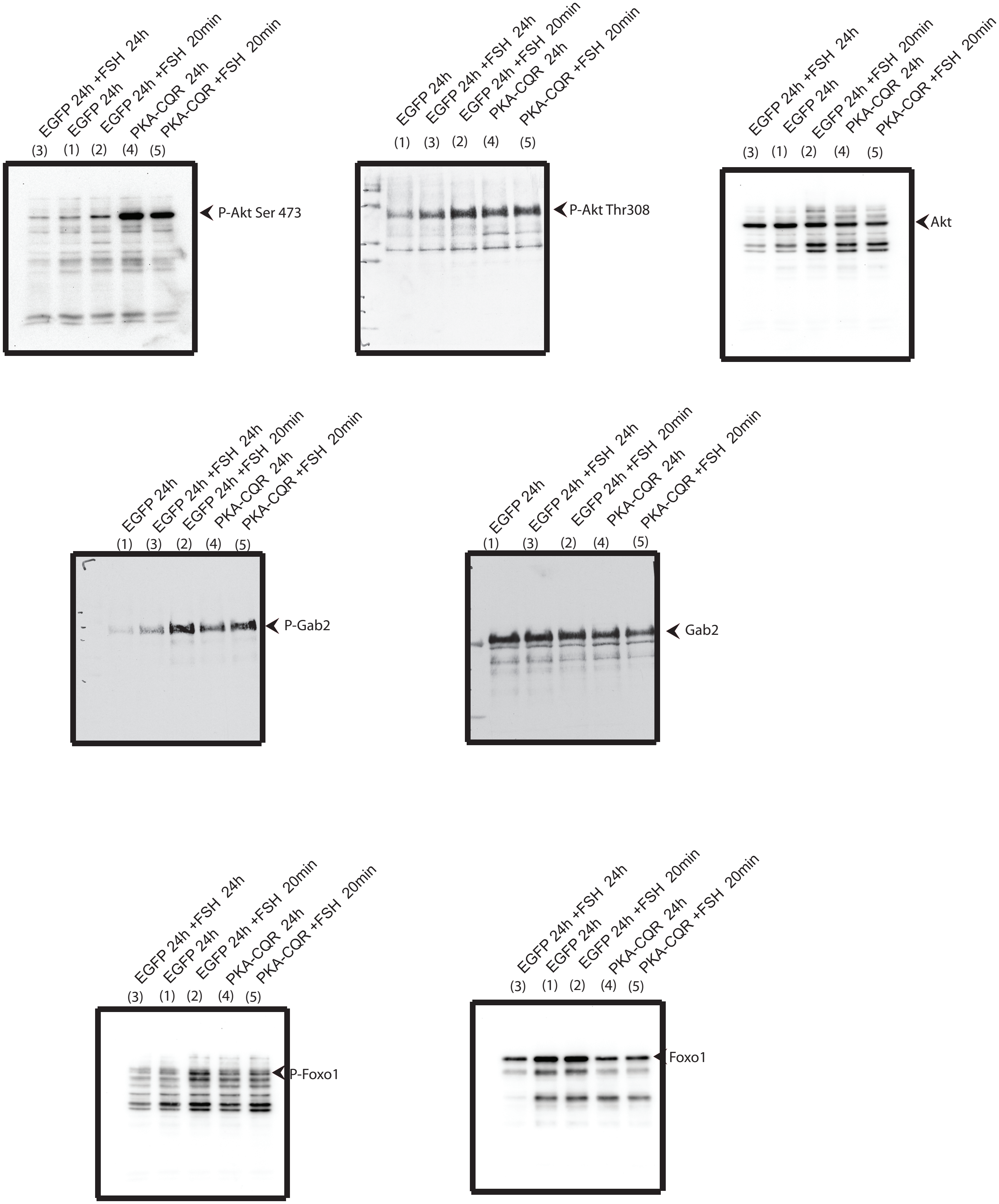

Supplementary Figure 3. Uncropped blots used in the construction of composite of Figure 3 in text. Numbers in parentheses correspond to lane identification numbers in Figure 3.

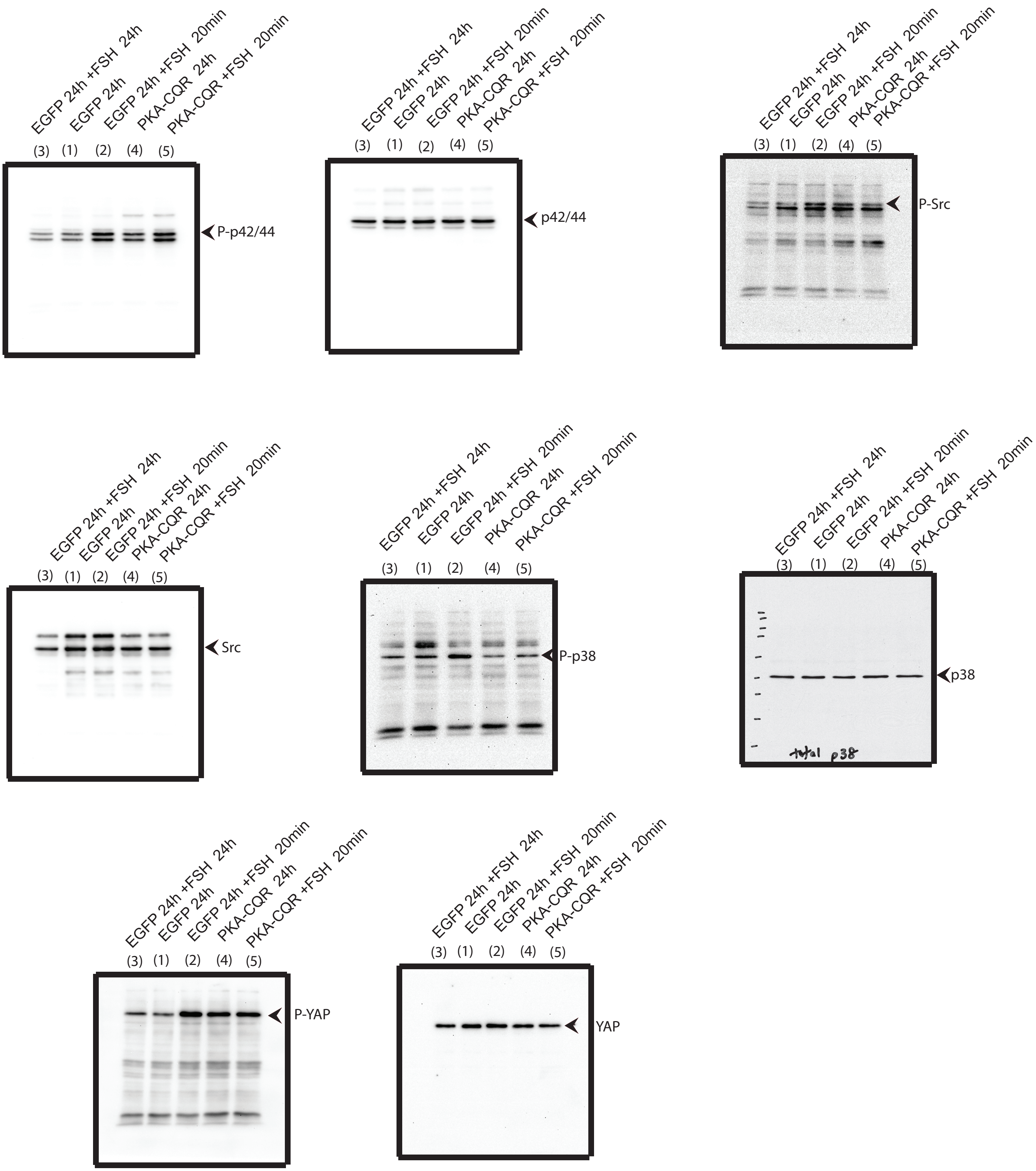

Supplementary Table S1. Transcripts increased or decreased > 2-fold by either FSH or PKA-CQR

ID	ENTREZ GENE NAME (Up-regulated Genes)	FSH/C	PKA-CQR/C	FSH/PKA
scd1	stearoyl-CoA desaturase (delta-9-desaturase)	18.184	2.005	9.07
vcam1	vascular cell adhesion molecule 1	8.652	1.734	4.99
rgcc	regulator of cell cycle	7.606	1.792	4.24
a2m	alpha-2-macroglobulin	5.191	1.440	3.60
RGD1565844	ring finger protein 138, retrogene 1	7.157	2.144	3.34
tmem150c	transmembrane protein 150C	4.956	1.495	3.32
defb24	defensin, beta 119	13.103	4.059	3.23
nfe2	nuclear factor, erythroid 2	12.116	3.947	3.07
ttyh2	tweety family member 2	5.340	1.884	2.83
eml1	echinoderm microtubule associated protein like 1	3.481	1.245	2.80
folr1	folate receptor 1 (adult)	4.255	1.545	2.75
hamp	hepcidin antimicrobial peptide	7.918	2.989	2.65
g0s2	G0/G1 switch 2	6.082	2.319	2.62
slc6a6	solute carrier family 6 (neurotransmitter transporter), member 6	7.040	2.703	2.60
slc7a8	solute carrier family 7 (amino acid transporter light chain, L system), member 8	3.694	1.425	2.59
adamts9	ADAM metallopeptidase with thrombospondin type 1 motif, 9	6.881	2.700	2.55
cxcr4	chemokine (C-X-C motif) receptor 4	13.944	5.487	2.54
kcnq5	potassium voltage-gated channel, KQT-like subfamily, member 5	3.356	1.328	2.53
crisp1	cysteine-rich secretory protein 1	2.931	1.195	2.45
hla-dra	major histocompatibility complex, class II, DR alpha	2.636	1.086	2.43
s100b	S100 calcium binding protein B	4.113	1.724	2.39
chst1	carbohydrate (keratan sulfate Gal-6) sulfotransferase 1	8.111	3.435	2.36
rbm47	RNA binding motif protein 47	13.385	5.716	2.34
ccl2	chemokine (C-C motif) ligand 2	2.507	1.079	2.32
exoc3l2	exocyst complex component 3-like 2	5.836	2.535	2.30
rnf125	ring finger protein 125, E3 ubiquitin protein ligase	2.216	0.964	2.30
fdxr	ferredoxin reductase	6.571	2.887	2.28
pdlim4	PDZ and LIM domain 4	2.952	1.304	2.26
apln	apelin	6.109	2.711	2.25
orm1	orosomucoid 1	2.583	1.149	2.25
susd3	sushi domain containing 3	4.260	1.912	2.23
pla2g1b	phospholipase A2, group IB (pancreas)	4.425	2.025	2.19
abcc9	ATP-binding cassette, sub-family C (CFTR/MRP), member 9	5.650	2.586	2.18
bpifb6	BPI fold containing family B, member 6	2.484	1.141	2.18
cbr3	carbonyl reductase 3	3.888	1.788	2.17
gas7	growth arrest-specific 7	2.669	1.232	2.17
chrnb4	cholinergic receptor, nicotinic, beta 4 (neuronal)	3.517	1.630	2.16
scn1b	sodium channel, voltage-gated, type I, beta subunit	2.220	1.042	2.13
crabp2	cellular retinoic acid binding protein 2	14.101	6.640	2.12
hoga1	4-hydroxy-2-oxoglutarate aldolase 1	2.366	1.120	2.11
lamb3	laminin, beta 3	3.334	1.590	2.10
hsd11b1	hydroxysteroid (11-beta) dehydrogenase 1	6.083	2.925	2.08
st8sia5	ST8 alpha-N-acetyl-neuraminide alpha-2,8-sialyltransferase 5	2.637	1.289	2.05
cd74	CD74 molecule, major histocompatibility complex, class II invariant chain	2.297	1.128	2.04
tmem86a	transmembrane protein 86A	11.081	5.558	1.99
eppin	EPPIN-WFDC6 readthrough	5.488	2.764	1.99
tspan33	tetraspanin 33	2.498	1.264	1.98
fam195a	family with sequence similarity 195, member A	4.862	2.469	1.97
mt2a	metallothionein 2A	4.664	2.372	1.97
tm7sf2	transmembrane 7 superfamily member 2	2.838	1.454	1.95

kcnq1	potassium voltage-gated channel, KQT-like subfamily, member 1	8.726	4.487	1.94
sgk1	serum/glucocorticoid regulated kinase 1	5.548	2.857	1.94
cldn11	claudin 11	5.075	2.641	1.92
id4	inhibitor of DNA binding 4, dominant negative helix-loop-helix protein	6.183	3.227	1.92
gprc5b	G protein-coupled receptor, class C, group 5, member B	3.015	1.582	1.91
reep1	receptor accessory protein 1	2.299	1.212	1.90
mmp19	matrix metallopeptidase 19	3.000	1.585	1.89
mro	maestro	7.587	4.058	1.87
tlcd1	TLC domain containing 1	2.697	1.446	1.87
mxra8	matrix-remodelling associated 8	2.001	1.078	1.86
cml1	N-acetyltransferase 8B (GCN5-related, putative, gene/pseudogene)	3.006	1.642	1.83
mvd	mevalonate (diphospho) decarboxylase	2.847	1.557	1.83
tmem178b	transmembrane protein 178B	4.387	2.400	1.83
nefh	neurofilament, heavy polypeptide	3.218	1.764	1.82
ubash3b	ubiquitin associated and SH3 domain containing B	3.380	1.853	1.82
dclk1	doublecortin-like kinase 1	2.063	1.133	1.82
ebp	emopamil binding protein (sterol isomerase)	2.183	1.201	1.82
rpp25	ribonuclease P/MRP 25kDa subunit	4.116	2.265	1.82
RGD1308117	chromosome 8 open reading frame 58	2.787	1.535	1.82
mrap	melanocortin 2 receptor accessory protein	10.419	5.781	1.80
gstm1	glutathione S-transferase mu 5	2.711	1.505	1.80
efnb1	ephrin-B1	2.815	1.568	1.80
gda	guanine deaminase	2.452	1.367	1.79
RGD1305464	chromosome 15 open reading frame 39	3.814	2.128	1.79
hopx	HOP homeobox	2.164	1.211	1.79
sgcg	sarcoglycan, gamma (35kDa dystrophin-associated glycoprotein)	2.648	1.484	1.78
n4bp2l1	NEDD4 binding protein 2-like 1	2.471	1.385	1.78
uhrf1bp1l	UHRF1 binding protein 1-like	2.708	1.531	1.77
agpat2	1-acylglycerol-3-phosphate O-acyltransferase 2	2.131	1.205	1.77
aacs	acetoacetyl-CoA synthetase	2.264	1.285	1.76
kcng2	potassium voltage-gated channel, subfamily G, member 2	3.097	1.760	1.76
ppl	periplakin	3.739	2.128	1.76
nt5e	5'-nucleotidase, ecto (CD73)	5.112	2.910	1.76
sepp1	selenoprotein P, plasma, 1	3.086	1.760	1.75
ppm1l	protein phosphatase, Mg2+/Mn2+ dependent, 1L	2.902	1.658	1.75
cyp4f4	cytochrome P450, family 4, subfamily F, polypeptide 8	3.229	1.848	1.75
mafb	v-maf avian musculoaponeurotic fibrosarcoma oncogene homolog B	2.823	1.620	1.74
hpgd	hydroxyprostaglandin dehydrogenase 15-(NAD)	4.152	2.390	1.74
rora	RAR-related orphan receptor A	2.148	1.240	1.73
aox1	aldehyde oxidase 1	3.590	2.075	1.73
timp3	TIMP metallopeptidase inhibitor 3	2.147	1.242	1.73
neu2	sialidase 2 (cytosolic sialidase)	2.078	1.209	1.72
ddit4	DNA-damage-inducible transcript 4	14.896	8.669	1.72
sema6d	sema domain, transmembrane domain (TM), and cytoplasmic domain, (semaphorin) 6D	3.443	2.005	1.72
kcnt1	potassium channel, subfamily T, member 1	3.137	1.830	1.71
ptpn5	protein tyrosine phosphatase, non-receptor type 5 (striatum-enriched)	2.148	1.255	1.71
cmbl	carboxymethylenebutenolidase homolog (Pseudomonas)	2.485	1.461	1.70
nppc	natriuretic peptide C	5.824	3.428	1.70
aldh1l1	aldehyde dehydrogenase 1 family, member L1	2.062	1.217	1.69
gstm7	glutathione S-transferase mu 2 (muscle)	4.114	2.430	1.69
cbfa2t3	core-binding factor, runt domain, alpha subunit 2; translocated to, 3	4.469	2.657	1.68
klhdc8a	kelch domain containing 8A	2.030	1.210	1.68
fam110b	family with sequence similarity 110, member B	2.023	1.206	1.68
nr0b2	nuclear receptor subfamily 0, group B, member 2	2.773	1.670	1.66
id2	inhibitor of DNA binding 2, dominant negative helix-loop-helix protein	2.308	1.394	1.66
entpd5	ectonucleoside triphosphate diphosphohydrolase 5	2.082	1.264	1.65

hsd11b2	hydroxysteroid (11-beta) dehydrogenase 2	3.511	2.134	1.65
cml5	camello-like 5	6.102	3.712	1.64
flvcr2	feline leukemia virus subgroup C cellular receptor family, member 2	3.682	2.248	1.64
notch1	notch 1	2.266	1.384	1.64
mir351	microRNA 351	5.162	3.154	1.64
pgap2	post-GPI attachment to proteins 2	2.311	1.413	1.64
fkbp5	FK506 binding protein 5	2.821	1.726	1.63
ass1	argininosuccinate synthase 1	18.488	11.350	1.63
enpp1	ectonucleotide pyrophosphatase/phosphodiesterase 1	2.030	1.249	1.63
unc79	unc-79 homolog (C. elegans)	3.361	2.068	1.63
upk1b	uroplakin 1B	4.040	2.488	1.62
map7d2	MAP7 domain containing 2	2.419	1.495	1.62
litaf	lipopolysaccharide-induced TNF factor	4.049	2.503	1.62
appbp2	amyloid beta precursor protein (cytoplasmic tail) binding protein 2	2.389	1.477	1.62
iqgap2	IQ motif containing GTPase activating protein 2	2.149	1.333	1.61
aldh3b1	aldehyde dehydrogenase 3 family, member B1	2.085	1.295	1.61
nqo1	NAD(P)H dehydrogenase, quinone 1	3.117	1.942	1.61
osgin1	oxidative stress induced growth inhibitor 1	17.605	10.993	1.60
rbks	ribokinase	2.193	1.372	1.60
dnajc15	DnaJ (Hsp40) homolog, subfamily C, member 15	2.760	1.727	1.60
clint1	clathrin interactor 1	2.200	1.379	1.60
mmd2	monocyte to macrophage differentiation-associated 2	7.769	4.870	1.60
tmem178a	transmembrane protein 178A	5.522	3.466	1.59
plac1	placenta-specific 1	3.486	2.197	1.59
adck3	aarF domain containing kinase 3	2.212	1.398	1.58
actr6	ARP6 actin-related protein 6 homolog (yeast)	2.509	1.586	1.58
b4galt6	UDP-Gal:betaGlcNAc beta 1,4- galactosyltransferase, polypeptide 6	3.001	1.901	1.58
rmdn2	regulator of microtubule dynamics 2	4.980	3.157	1.58
mob4	MOB family member 4, phocein	3.052	1.947	1.57
soat1	sterol O-acyltransferase 1	2.585	1.650	1.57
reep2	receptor accessory protein 2	3.066	1.959	1.57
inmt	indolethylamine N-methyltransferase	24.402	15.640	1.56
star	steroidogenic acute regulatory protein	14.132	9.110	1.55
parm1	prostate androgen-regulated mucin-like protein 1	6.033	3.908	1.54
ccdc69	coiled-coil domain containing 69	2.997	1.942	1.54
calml3	calmodulin-like 3	10.546	6.842	1.54
aldh2	aldehyde dehydrogenase 2 family (mitochondrial)	2.422	1.574	1.54
fbxo16	F-box protein 16	2.169	1.412	1.54
scara5	scavenger receptor class A, member 5 (putative)	7.304	4.757	1.54
dcxr	dicarbonyl/L-xylulose reductase	2.411	1.580	1.53
c1qtnf1	C1q and tumor necrosis factor related protein 1	2.209	1.455	1.52
pc	pyruvate carboxylase	2.144	1.416	1.51
rab3a	RAB3A, member RAS oncogene family	2.218	1.466	1.51
lss	lanosterol synthase (2,3-oxidosqualene-lanosterol cyclase)	2.137	1.418	1.51
gramd1b	GRAM domain containing 1B	3.233	2.147	1.51
fads2	fatty acid desaturase 2	2.500	1.661	1.51
wwp2	WW domain containing E3 ubiquitin protein ligase 2	3.864	2.589	1.49
fam69b	family with sequence similarity 69, member B	2.165	1.452	1.49
mvk	mevalonate kinase	2.105	1.414	1.49
fbxo2	F-box protein 2	2.520	1.697	1.48
hist1h4h	histone cluster 1, H4h	2.604	1.754	1.48
spin2a	spindlin family, member 2C	2.648	1.790	1.48
slfn3	schlafen family member 12	3.289	2.224	1.48
uxs1	UDP-glucuronate decarboxylase 1	2.558	1.731	1.48
nudt10	nudix (nucleoside diphosphate linked moiety X)-type motif 11	2.172	1.473	1.47

tfrc	transferrin receptor	2.526	1.717	1.47
smco4	single-pass membrane protein with coiled-coil domains 4	2.243	1.526	1.47
ptplb	protein tyrosine phosphatase-like (proline instead of catalytic arginine), member b	2.557	1.748	1.46
sphk1	sphingosine kinase 1	2.573	1.765	1.46
slc16a6	solute carrier family 16, member 6	3.632	2.496	1.46
gal	galanin/GMAP prepropeptide	5.008	3.444	1.45
adam8	ADAM metallopeptidase domain 8	2.013	1.388	1.45
RGD1563510	similar to RIKEN cDNA 8430427H17 gene	2.078	1.435	1.45
ier5l	immediate early response 5-like	2.020	1.397	1.45
gsta3	glutathione S-transferase alpha 3	2.531	1.754	1.44
sbk1	SH3 domain binding kinase 1	2.767	1.919	1.44
mt3	metallothionein 3	2.129	1.479	1.44
abca1	ATP-binding cassette, sub-family A (ABC1), member 1	2.262	1.573	1.44
adam19	ADAM metallopeptidase domain 19	2.779	1.940	1.43
reep6	receptor accessory protein 6	2.840	1.986	1.43
xpnpep2	X-prolyl aminopeptidase (aminopeptidase P) 2, membrane-bound	2.270	1.589	1.43
clcf1	cardiotrophin-like cytokine factor 1	3.609	2.536	1.42
tgfbi	transforming growth factor, beta-induced, 68kDa	3.860	2.714	1.42
leprel4	leprecan-like 4	2.001	1.416	1.41
il18	interleukin 18	4.042	2.864	1.41
cyp11a1	cytochrome P450, family 11, subfamily A, polypeptide 1	51.313	36.405	1.41
gstm5	glutathione S-transferase mu 3 (brain)	2.010	1.427	1.41
fdft1	farnesyl-diphosphate farnesyltransferase 1	2.826	2.007	1.41
gale	UDP-galactose-4-epimerase	2.500	1.780	1.40
fam122b	family with sequence similarity 122B	3.346	2.390	1.40
gch1	GTP cyclohydrolase 1	9.003	6.437	1.40
aig1	androgen-induced 1	2.156	1.542	1.40
epha2	EPH receptor A2	2.126	1.522	1.40
galnt7	polypeptide N-acetylgalactosaminyltransferase 7	2.126	1.524	1.40
cgnl1	cingulin-like 1	3.923	2.813	1.39
id1	inhibitor of DNA binding 1, dominant negative helix-loop-helix protein	2.852	2.050	1.39
phyh	phytanoyl-CoA 2-hydroxylase	2.286	1.647	1.39
mapkapk3	mitogen-activated protein kinase-activated protein kinase 3	2.231	1.608	1.39
gamt	guanidinoacetate N-methyltransferase	2.016	1.454	1.39
tsx	testis specific X-linked gene	2.046	1.476	1.39
irak2	interleukin-1 receptor-associated kinase 2	3.532	2.552	1.38
fuca1	fucosidase, alpha-L- 1, tissue	2.441	1.764	1.38
sfxn5	sideroflexin 5	2.160	1.565	1.38
baiap2	BAI1-associated protein 2	2.618	1.897	1.38
cotl1	coactosin-like F-actin binding protein 1	11.008	7.983	1.38
sh3tc2	SH3 domain and tetratricopeptide repeats 2	2.066	1.499	1.38
exoc3l1	exocyst complex component 3-like 1	2.064	1.499	1.38
lcn2	lipocalin 2	3.111	2.261	1.38
acsl3	acyl-CoA synthetase long-chain family member 3	2.137	1.562	1.37
LOC363060		2.175	1.591	1.37
lpcat3	lysophosphatidylcholine acyltransferase 3	2.488	1.823	1.36
tekt1	tektin 1	2.332	1.710	1.36
dlgap1	discs, large (Drosophila) homolog-associated protein 1	2.008	1.474	1.36
bdh1	3-hydroxybutyrate dehydrogenase, type 1	2.001	1.476	1.36
anp32e	acidic (leucine-rich) nuclear phosphoprotein 32 family, member E	2.423	1.789	1.35
msmo1	methylsterol monooxygenase 1	2.020	1.493	1.35
wipf3	WAS/WASL interacting protein family, member 3	2.159	1.596	1.35
slc25a1	solute carrier family 25 (mitochondrial carrier, citrate transporter), member 1	2.099	1.553	1.35
slc25a21	solute carrier family 25 (mitochondrial oxoadipate carrier), member 21	4.359	3.226	1.35
epb41l1	erythrocyte membrane protein band 4.1-like 1	2.118	1.570	1.35
il4r	interleukin 4 receptor	2.260	1.677	1.35

hpcal1	hippocalcin-like 1	2.037	1.513	1.35
LOC688328		3.868	2.880	1.34
msi2	musashi RNA-binding protein 2	2.434	1.816	1.34
scarb1	scavenger receptor class B, member 1	25.153	18.772	1.34
pcsk9	proprotein convertase subtilisin/kexin type 9	2.150	1.608	1.34
prlr	prolactin receptor	5.444	4.084	1.33
fam102a	family with sequence similarity 102, member A	2.011	1.510	1.33
cybb	cytochrome b-245, beta polypeptide	2.988	2.255	1.33
slc25a30	solute carrier family 25, member 30	5.561	4.199	1.32
dhcr7	7-dehydrocholesterol reductase	2.860	2.163	1.32
lmo2	LIM domain only 2 (rhombotin-like 1)	2.919	2.208	1.32
npas2	neuronal PAS domain protein 2	7.096	5.368	1.32
fhdc1	FH2 domain containing 1	2.098	1.589	1.32
cxcl12	chemokine (C-X-C motif) ligand 12	2.444	1.855	1.32
sod2	superoxide dismutase 2, mitochondrial	2.733	2.080	1.31
aadat	aminoadipate aminotransferase	3.434	2.614	1.31
acat2	acetyl-CoA acetyltransferase 2	2.261	1.723	1.31
nt5c3b	5'-nucleotidase, cytosolic IIIB	2.008	1.536	1.31
pla2g4a	phospholipase A2, group IVA (cytosolic, calcium-dependent)	4.061	3.119	1.30
cobll1	cordon-bleu WH2 repeat protein-like 1	2.026	1.558	1.30
isyna1	inositol-3-phosphate synthase 1	3.369	2.592	1.30
runx1	runt-related transcription factor 1	3.459	2.687	1.29
nkain3	Na+/K+ transporting ATPase interacting 3	2.071	1.610	1.29
hspd1	heat shock 60kDa protein 1 (chaperonin)	2.350	1.832	1.28
reg3g	regenerating islet-derived 3 gamma	2.437	1.900	1.28
snapc1	small nuclear RNA activating complex, polypeptide 1, 43kDa	2.097	1.639	1.28
fasn	fatty acid synthase	2.135	1.670	1.28
LOC100359971	hypothetical protein LOC100359971	2.309	1.810	1.28
herpud1	homocysteine-inducible, endoplasmic reticulum stress-inducible, ubiquitin-like domain member 1	2.329	1.827	1.27
LOC689176	similar to transmembrane protein 64	2.870	2.255	1.27
dusp15	dual specificity phosphatase 15	2.025	1.592	1.27
abhd13	abhydrolase domain containing 13	2.491	1.966	1.27
srebf1	sterol regulatory element binding transcription factor 1	2.277	1.800	1.27
csdc2	cold shock domain containing C2, RNA binding	2.501	1.982	1.26
hmgcs1	3-hydroxy-3-methylglutaryl-CoA synthase 1 (soluble)	2.232	1.769	1.26
net1	neuroepithelial cell transforming 1	3.361	2.669	1.26
nfkbib	nuclear factor of kappa light polypeptide gene enhancer in B-cells inhibitor, beta	2.052	1.631	1.26
mfge8	milk fat globule-EGF factor 8 protein	2.019	1.607	1.26
lppr5	lipid phosphate phosphatase-related protein type 5	2.399	1.917	1.25
nr5a2	nuclear receptor subfamily 5, group A, member 2	2.350	1.884	1.25
hspe1	heat shock 10kDa protein 1	2.340	1.887	1.24
stard4	StAR-related lipid transfer (START) domain containing 4	2.470	1.992	1.24
plxnc1	plexin C1	3.342	2.698	1.24
sqle	squalene epoxidase	2.423	1.960	1.24
fzd8	frizzled class receptor 8	2.156	1.745	1.24
rnase4	ribonuclease, RNase A family, 4	2.301	1.870	1.23
nr1h3	nuclear receptor subfamily 1, group H, member 3	2.440	1.983	1.23
fam46c	family with sequence similarity 46, member C	3.810	3.100	1.23
filip1	filamin A interacting protein 1	4.034	3.286	1.23
etnk2	ethanolamine kinase 2	3.136	2.556	1.23
madd	MAP-kinase activating death domain	2.099	1.733	1.21
fam110c	family with sequence similarity 110, member C	38.404	31.807	1.21
micall2	MICAL-like 2	2.848	2.372	1.20
ptplad1	protein tyrosine phosphatase-like A domain containing 1	3.308	2.757	1.20
cx3cl1	chemokine (C-X3-C motif) ligand 1	2.368	1.975	1.20
efhd1	EF-hand domain family, member D1	2.241	1.870	1.20

gfpt2	glutamine-fructose-6-phosphate transaminase 2	5.369	4.482	1.20
creb3l1	cAMP responsive element binding protein 3-like 1	3.667	3.069	1.19
tns4	tensin 4	2.418	2.037	1.19
hist1h3h	histone cluster 1, H3h	2.189	1.851	1.18
por	P450 (cytochrome) oxidoreductase	2.301	1.951	1.18
map7	microtubule-associated protein 7	2.234	1.903	1.17
crispld2	cysteine-rich secretory protein LCCL domain containing 2	2.445	2.092	1.17
tle3	transducin-like enhancer of split 3	2.114	1.814	1.17
me2	malic enzyme 2, NAD(+)-dependent, mitochondrial	2.240	1.927	1.16
itga1	integrin, alpha 1	2.164	1.866	1.16
rasd1	RAS, dexamethasone-induced 1	6.524	5.669	1.15
ccl5	chemokine (C-C motif) ligand 5	3.064	2.666	1.15
nr5a1	nuclear receptor subfamily 5, group A, member 1	2.356	2.051	1.15
btbd11	BTB (POZ) domain containing 11	3.000	2.622	1.14
cyp51	cytochrome P450, family 51, subfamily A, polypeptide 1	2.127	1.861	1.14
cebpb	CCAAT/enhancer binding protein (C/EBP), beta	2.020	1.769	1.14
per1	period circadian clock 1	2.143	1.880	1.14
aldh1a3	aldehyde dehydrogenase 1 family, member A3	3.873	3.404	1.14
idi1	isopentenyl-diphosphate delta isomerase 1	2.783	2.446	1.14
phactr1	phosphatase and actin regulator 1	2.074	1.833	1.13
adipor2	adiponectin receptor 2	2.157	1.908	1.13
mageb16	melanoma antigen family B, 16	3.454	3.058	1.13
ifi27l2b	interferon, alpha-inducible protein 27 like 2B	2.066	1.849	1.12
hunk	hormonally up-regulated Neu-associated kinase	2.361	2.123	1.11
inhba	inhibin, beta A	2.765	2.501	1.11
fdx1	ferredoxin 1	8.319	7.526	1.11
sox18	SRY (sex determining region Y)-box 18	2.920	2.642	1.11
cd40	CD40 molecule, TNF receptor superfamily member 5	2.343	2.126	1.10
st3gal4	ST3 beta-galactoside alpha-2,3-sialyltransferase 4	2.304	2.091	1.10
alas1	aminolevulinate, delta-, synthase 1	7.027	6.384	1.10
rps6ka5	ribosomal protein S6 kinase, polypeptide 5	2.513	2.286	1.10
rasal1	RAS protein activator like 1 (GAP1 like)	2.790	2.540	1.10
brd8	bromodomain containing 8	2.688	2.449	1.10
RGD1561113	chromosome 9 open reading frame 16	2.276	2.074	1.10
hsd17b7	hydroxysteroid (17-beta) dehydrogenase 7	2.841	2.592	1.10
thrb	thyroid hormone receptor, beta	2.248	2.051	1.10
slc22a23	solute carrier family 22, member 23	2.099	1.916	1.10
bcat1	branched chain amino-acid transaminase 1, cytosolic	2.378	2.190	1.09
slc25a28	solute carrier family 25 (mitochondrial iron transporter), member 28	2.002	1.857	1.08
gde1	glycerophosphodiester phosphodiesterase 1	2.363	2.206	1.07
gclc	glutamate-cysteine ligase, catalytic subunit	2.012	1.880	1.07
tmem97	transmembrane protein 97	2.471	2.309	1.07
nabp1	nucleic acid binding protein 1	2.047	1.914	1.07
srxn1	sulfiredoxin 1	3.015	2.850	1.06
enc1	ectodermal-neural cortex 1 (with BTB domain)	4.896	4.635	1.06
ptgds	prostaglandin D2 synthase 21kDa (brain)	13.259	12.594	1.05
acsbg1	acyl-CoA synthetase bubblegum family member 1	2.564	2.455	1.04
lama4	laminin, alpha 4	2.139	2.053	1.04
ext1	exostosin glycosyltransferase 1	2.166	2.079	1.04
cxcr7	atypical chemokine receptor 3	2.925	2.810	1.04
nefm	neurofilament, medium polypeptide	2.052	1.976	1.04
hey2	hes-related family bHLH transcription factor with YRPW motif 2	4.250	4.138	1.03
gata4	GATA binding protein 4	2.307	2.251	1.02
inhbb	inhibin, beta B	3.435	3.353	1.02
inha	inhibin, alpha	2.431	2.406	1.01
pcdh18	protocadherin 18	3.367	3.342	1.01

prss35	protease, serine, 35	2.147	2.134	1.01
galnt6	polypeptide N-acetylgalactosaminyltransferase 6	2.117	2.112	1.00
egfr	epidermal growth factor receptor	2.034	2.043	1.00
gjb2	gap junction protein, beta 2, 26kDa	6.820	6.872	0.99
tmem45a	transmembrane protein 45A	2.072	2.115	0.98
fam43a	family with sequence similarity 43, member A	2.016	2.069	0.97
Orc1	origin recognition complex, subunit 1	1.956	2.025	0.97
car14	carbonic anhydrase XIV	2.337	2.441	0.96
kcne4	potassium voltage-gated channel, Isk-related family, member 4	4.184	4.416	0.95
giot1	gonadotropin inducible ovarian transcription factor 1	5.711	6.072	0.94
gdf11	growth differentiation factor 11	1.931	2.057	0.94
LOC100910792	amphiphysin	3.053	3.299	0.93
hsd17b1	hydroxysteroid (17-beta) dehydrogenase 1	3.218	3.481	0.92
rrm2	ribonucleotide reductase M2	2.000	2.167	0.92
oasl	2'-5'-oligoadenylate synthetase-like	2.044	2.220	0.92
cldn10	claudin 10	7.175	7.800	0.92
Ccrn4l	CCR4 carbon catabolite repression 4-like (S. cerevisiae)	1.948	2.120	0.92
vcan	versican	2.053	2.238	0.92
rgs2	regulator of G-protein signaling 2, 24kDa	3.479	3.793	0.92
Ccnd2	cyclin D2	1.937	2.120	0.91
clmp	CXADR-like membrane protein	3.627	3.971	0.91
dync1i1	dynein, cytoplasmic 1, intermediate chain 1	2.344	2.579	0.91
plau	plasminogen activator, urokinase	2.244	2.475	0.91
chst2	carbohydrate (N-acetylglucosamine-6-O) sulfotransferase 2	2.577	2.901	0.89
grk5	G protein-coupled receptor kinase 5	3.056	3.476	0.88
dyrk3	dual-specificity tyrosine-(Y)-phosphorylation regulated kinase 3	2.627	3.031	0.87
sik1	salt inducible kinase 1	2.248	2.598	0.87
chst8	carbohydrate (N-acetylgalactosamine 4-0) sulfotransferase 8	3.028	3.533	0.86
cthrc1	collagen triple helix repeat containing 1	2.053	2.411	0.85
dcaf12	DDB1 and CUL4 associated factor 12-like 1	2.065	2.431	0.85
ihh	indian hedgehog	2.363	2.808	0.84
crem	cAMP responsive element modulator	3.300	3.966	0.83
kif22	kinesin family member 22	2.172	2.615	0.83
Prkcb	protein kinase C, beta	1.694	2.050	0.83
nup62cl	nucleoporin 62 C-terminal like	3.255	4.009	0.81
Clic3	chloride intracellular channel 3	1.819	2.243	0.81
asphd2	aspartate beta-hydroxylase domain containing 2	2.329	2.880	0.81
Plekhd1	pleckstrin homology domain containing, family D (with coiled-coil domains) member 1	1.812	2.249	0.81
nr4a1	nuclear receptor subfamily 4, group A, member 1	3.114	3.869	0.80
Msh2	mutS homolog 2	1.612	2.030	0.79
Cd55	CD55 molecule, decay accelerating factor for complement (Cromer blood group)	1.978	2.513	0.79
gmnn	geminin, DNA replication inhibitor	3.456	4.518	0.76
Ns5atp9	KIAA0101	1.707	2.234	0.76
Kif20a	kinesin family member 20A	1.991	2.615	0.76
cxcl13	chemokine (C-X-C motif) ligand 13	2.773	3.655	0.76
Pde4d	phosphodiesterase 4D, cAMP-specific	1.838	2.435	0.75
ephb3	EPH receptor B3	2.641	3.552	0.74
Angptl1	angiopoietin-like 1	1.862	2.509	0.74
osgin2	oxidative stress induced growth inhibitor family member 2	5.898	8.088	0.73
E2f8	E2F transcription factor 8	1.500	2.062	0.73
Cdt1	chromatin licensing and DNA replication factor 1	1.569	2.196	0.71
Isg15	ISG15 ubiquitin-like modifier	1.527	2.189	0.70
sult1b1	sulfotransferase family, cytosolic, 1B, member 1	8.839	12.683	0.70
lhcgr	luteinizing hormone/choriogonadotropin receptor	27.986	41.788	0.67
Ednrb	endothelin receptor type B	1.333	2.018	0.66

grem2	gremlin 2, DAN family BMP antagonist	3.198	4.890	0.65
tnfaip6	tumor necrosis factor, alpha-induced protein 6	30.148	46.638	0.65
cftr	cystic fibrosis transmembrane conductance regulator (ATP-binding cassette sub-family C, member 7)	2.248	3.484	0.65
plat	plasminogen activator, tissue	4.737	7.353	0.64
Dio2	deiodinase, iodothyronine, type II	1.389	2.179	0.64
pde4b	phosphodiesterase 4B, cAMP-specific	17.981	28.364	0.63
Snap25	synaptosomal-associated protein 25Kda	1.601	2.574	0.62
Plk2	polo-like kinase 2	1.446	2.335	0.62
Sord	sorbitol dehydrogenase	1.659	2.918	0.57
adcyap1	adenylate cyclase activating polypeptide 1 (pituitary)	2.964	5.229	0.57
Car2	carbonic anhydrase II	1.374	2.551	0.54
Tmem252	transmembrane protein 252	1.505	2.811	0.54
cyp19a1	cytochrome P450, family 19, subfamily A, polypeptide 1	29.441	59.709	0.49
nap1l5	nucleosome assembly protein 1-like 5	8.370	17.313	0.48
Slc7a11	solute carrier family 7, member 11	1.337	2.896	0.46
Havcr1	hepatitis A virus cellular receptor 1	0.947	2.095	0.45
Nr4a3	nuclear receptor subfamily 4, group A, member 3	1.805	4.200	0.43
Cga	glycoprotein hormones, alpha polypeptide	1.807	4.397	0.41
Npy1r	neuropeptide Y receptor Y1	1.973	4.826	0.41
Pgr	progesterone receptor	1.745	4.295	0.41
akr1b8	aldo-keto reductase family 1, member B10 (aldose reductase)	3.179	7.965	0.40
fabp5	fatty acid binding protein 5 (psoriasis-associated)	2.571	6.452	0.40
Rgs1	regulator of G-protein signaling 1	1.335	3.413	0.39
ptgs2	prostaglandin-endoperoxide synthase 2 (prostaglandin G/H synthase and cyclooxygenase)	12.770	35.200	0.36
scd	stearoyl-Coenzyme A desaturase 2	2.132	5.995	0.36
ereg	epiregulin	5.369	18.101	0.30
gja5	gap junction protein, alpha 5, 40kDa	4.663	16.257	0.29
areg	amphiregulin	3.572	13.836	0.26
**ID**	**ENTREZ GENE NAME (Down-regulated Genes)**	**C /FSH**	**C/PKA-CQR**	**FSH/PKA**
pamr1	peptidase domain containing associated with muscle regeneration 1	-49.126	-3.030	0.06
tnfrsf11b	tumor necrosis factor receptor superfamily, member 11b	-65.433	-5.059	0.08
fam65b	family with sequence similarity 65, member B	-27.998	-3.010	0.11
anpep	alanyl (membrane) aminopeptidase	-20.897	-2.373	0.11
rcan2	regulator of calcineurin 2	-10.057	-1.242	0.12
adamts5	ADAM metallopeptidase with thrombospondin type 1 motif, 5	-17.976	-2.284	0.13
agtr2	angiotensin II receptor, type 2	-32.295	-4.116	0.13
aspn	asporin	-21.456	-2.862	0.13
arhgap6	Rho GTPase activating protein 6	-14.076	-2.005	0.14
lox	lysyl oxidase	-12.061	-1.825	0.15
pdlim3	PDZ and LIM domain 3	-21.345	-3.725	0.17
agtr1a	angiotensin II receptor, type 1	-21.200	-4.450	0.21
lmcd1	LIM and cysteine-rich domains 1	-19.922	-4.417	0.22
cdh11	cadherin 11, type 2, OB-cadherin (osteoblast)	-19.393	-4.352	0.22
nov	nephroblastoma overexpressed	-7.104	-1.633	0.23
mfap5	microfibrillar associated protein 5	-9.731	-2.253	0.23
zcchc18	zinc finger, CCHC domain containing 18	-6.376	-1.528	0.24
fn1	fibronectin 1	-10.737	-2.597	0.24
p4ha3	prolyl 4-hydroxylase, alpha polypeptide III	-20.228	-4.969	0.25
cdo1	cysteine dioxygenase type 1	-12.258	-3.078	0.25
tagln	transgelin	-15.064	-3.866	0.26
ifitm1	interferon induced transmembrane protein 1	-8.250	-2.119	0.26
sybu	syntabulin (syntaxin-interacting)	-5.469	-1.415	0.26
cyp26b1	cytochrome P450, family 26, subfamily B, polypeptide 1	-6.693	-1.769	0.26
klf5	Kruppel-like factor 5 (intestinal)	-15.351	-4.086	0.27

pdk4	pyruvate dehydrogenase kinase, isozyme 4	-10.918	-2.924	0.27
col11a1	collagen, type XI, alpha 1	-5.845	-1.588	0.27
pcdh19	protocadherin 19	-3.469	-0.973	0.28
bdnf	brain-derived neurotrophic factor	-13.213	-3.779	0.29
dkk3	dickkopf WNT signaling pathway inhibitor 3	-6.044	-1.739	0.29
rassf4	Ras association (RalGDS/AF-6) domain family member 4	-5.787	-1.725	0.30
lrrfip1	leucine rich repeat (in FLII) interacting protein 1	-6.875	-2.067	0.30
nr0b1	nuclear receptor subfamily 0, group B, member 1	-5.932	-1.829	0.31
xdh	xanthine dehydrogenase	-6.348	-2.008	0.32
scn4b	sodium channel, voltage-gated, type IV, beta subunit	-2.607	-0.848	0.33
nxnl2	nucleoredoxin-like 2	-3.471	-1.137	0.33
ptges	prostaglandin E synthase	-2.902	-0.957	0.33
rapgef5	Rap guanine nucleotide exchange factor (GEF) 5	-4.450	-1.475	0.33
hrct1	histidine rich carboxyl terminus 1	-5.601	-1.873	0.33
cyr61	cysteine-rich, angiogenic inducer, 61	-10.668	-3.617	0.34
spon1	spondin 1, extracellular matrix protein	-2.552	-0.877	0.34
sorbs1	sorbin and SH3 domain containing 1	-10.278	-3.581	0.35
popdc2	popeye domain containing 2	-7.931	-2.778	0.35
kcnk2	potassium channel, subfamily K, member 2	-4.033	-1.429	0.35
lims2	LIM and senescent cell antigen-like domains 2	-18.396	-6.532	0.36
sytl2	synaptotagmin-like 2	-7.234	-2.605	0.36
fry	furry homolog (Drosophila)	-3.380	-1.221	0.36
ehd4	EH-domain containing 4	-6.173	-2.251	0.36
atoh8	atonal homolog 8 (Drosophila)	-4.205	-1.541	0.37
lhfp	lipoma HMGIC fusion partner	-6.795	-2.493	0.37
irx3	iroquois homeobox 3	-4.883	-1.804	0.37
cidea	cell death-inducing DFFA-like effector a	-4.669	-1.728	0.37
mcam	melanoma cell adhesion molecule	-5.335	-1.980	0.37
fgf18	fibroblast growth factor 18	-5.195	-1.952	0.38
filip1l	filamin A interacting protein 1-like	-4.738	-1.809	0.38
cav2	caveolin 2	-10.074	-3.858	0.38
fam181b	family with sequence similarity 181, member B	-3.585	-1.373	0.38
ydjc	YdjC homolog (bacterial)	-6.100	-2.340	0.38
tex14	testis expressed 14	-5.622	-2.161	0.38
coch	cochlin	-3.618	-1.394	0.39
limch1	LIM and calponin homology domains 1	-2.766	-1.067	0.39
tgfb2	transforming growth factor, beta 2	-7.033	-2.714	0.39
serping1	serpin peptidase inhibitor, clade G (C1 inhibitor), member 1	-6.239	-2.418	0.39
nid1	nidogen 1	-5.972	-2.331	0.39
dcn	decorin	-4.563	-1.790	0.39
ptplad2	protein tyrosine phosphatase-like A domain containing 2	-4.295	-1.688	0.39
meox2	mesenchyme homeobox 2	-12.977	-5.140	0.40
cdc42ep3	CDC42 effector protein (Rho GTPase binding) 3	-8.467	-3.366	0.40
spp1	secreted phosphoprotein 1	-5.676	-2.270	0.40
mgp	matrix Gla protein	-2.850	-1.143	0.40
pgm2l1	phosphoglucomutase 2-like 1	-4.461	-1.795	0.40
ptger3	prostaglandin E receptor 3 (subtype EP3)	-5.398	-2.175	0.40
angpt2	angiopoietin 2	-4.724	-1.928	0.41
rnf182	ring finger protein 182	-4.836	-2.007	0.42
hook1	hook microtubule-tethering protein 1	-3.315	-1.377	0.42
cav1	caveolin 1, caveolae protein, 22kDa	-6.089	-2.559	0.42
cbln1	cerebellin 1 precursor	-2.534	-1.072	0.42
cald1	caldesmon 1	-9.697	-4.145	0.43
htra2	HtrA serine peptidase 2	-2.502	-1.075	0.43
crim1	cysteine rich transmembrane BMP regulator 1 (chordin-like)	-4.597	-1.986	0.43

slc16a7	solute carrier family 16 (monocarboxylate transporter), member 7	-4.587	-1.990	0.43
arnt2	aryl-hydrocarbon receptor nuclear translocator 2	-5.966	-2.657	0.45
lpar1	lysophosphatidic acid receptor 1	-6.300	-2.811	0.45
otud1	OTU deubiquitinase 1	-5.878	-2.627	0.45
clu	clusterin	-3.626	-1.622	0.45
akap12	A kinase (PRKA) anchor protein 12	-7.955	-3.566	0.45
rgma	repulsive guidance molecule family member a	-3.307	-1.483	0.45
tspan8	tetraspanin 8	-6.199	-2.781	0.45
apcdd1	adenomatosis polyposis coli down-regulated 1	-3.312	-1.488	0.45
adm	adrenomedullin	-8.220	-3.713	0.45
tdrd7	tudor domain containing 7	-4.721	-2.140	0.45
ryr2	ryanodine receptor 2 (cardiac)	-3.291	-1.497	0.45
tfcp2l1	transcription factor CP2-like 1	-3.378	-1.541	0.46
kif23	kinesin family member 23	-2.322	-1.077	0.46
runx1t1	runt-related transcription factor 1; translocated to, 1 (cyclin D-related)	-8.384	-3.889	0.46
ets1	v-ets avian erythroblastosis virus E26 oncogene homolog 1	-6.238	-2.916	0.47
serpinf1	serpin peptidase inhibitor, clade F (alpha-2 antiplasmin, pigment epithelium derived factor), member 1	-2.396	-1.121	0.47
ppp1r3c	protein phosphatase 1, regulatory subunit 3C	-4.658	-2.198	0.47
pik3ip1	phosphoinositide-3-kinase interacting protein 1	-3.485	-1.647	0.47
sqrdl	sulfide quinone reductase-like (yeast)	-4.214	-1.992	0.47
kcnab1	potassium voltage-gated channel, shaker-related subfamily, beta member 1	-2.198	-1.046	0.48
procr	protein C receptor, endothelial	-2.237	-1.067	0.48
prss23	protease, serine, 23	-2.927	-1.398	0.48
pawr	PRKC, apoptosis, WT1, regulator	-3.825	-1.827	0.48
LOC100363275	G protein-coupled receptor 124	-3.091	-1.477	0.48
sspn	sarcospan	-3.604	-1.749	0.49
ccdc3	coiled-coil domain containing 3	-3.125	-1.519	0.49
me3	malic enzyme 3, NADP(+)-dependent, mitochondrial	-3.184	-1.550	0.49
fam84a	family with sequence similarity 84, member A	-4.677	-2.277	0.49
ppp1r14a	protein phosphatase 1, regulatory (inhibitor) subunit 14A	-2.903	-1.422	0.49
mid1	midline 1 (Opitz/BBB syndrome)	-4.857	-2.393	0.49
got1l1	glutamic-oxaloacetic transaminase 1-like 1	-3.667	-1.820	0.50
dmd	dystrophin	-2.047	-1.026	0.50
kcnk6	potassium channel, subfamily K, member 6	-4.185	-2.121	0.51
hbq1b	hemoglobin, theta 1	-2.573	-1.305	0.51
diaph3	diaphanous-related formin 3	-2.639	-1.340	0.51
wwc2	WW and C2 domain containing 2	-2.573	-1.309	0.51
mcart1l	solute carrier family 25, member 53	-2.260	-1.157	0.51
RGD1561724	similar to mKIAA0716 protein	-3.249	-1.665	0.51
cys1	cystin 1	-3.078	-1.592	0.52
LOC685067	guanylate binding protein 7	-2.432	-1.269	0.52
cdc42ep2	CDC42 effector protein (Rho GTPase binding) 2	-3.087	-1.621	0.53
sik2	salt-inducible kinase 2	-2.713	-1.425	0.53
vwa1	von Willebrand factor A domain containing 1	-3.322	-1.747	0.53
cd83	CD83 molecule	-4.058	-2.136	0.53
slco3a1	solute carrier organic anion transporter family, member 3A1	-2.402	-1.266	0.53
omd	osteomodulin	-2.276	-1.212	0.53
myo1d	myosin ID	-3.516	-1.874	0.53
mboat1	membrane bound O-acyltransferase domain containing 1	-3.174	-1.695	0.53
fam46a	family with sequence similarity 46, member A	-3.008	-1.613	0.54
calhm2	calcium homeostasis modulator 2	-3.571	-1.917	0.54
rbpms	RNA binding protein with multiple splicing	-3.166	-1.705	0.54
ccdc109b	coiled-coil domain containing 109B	-2.324	-1.254	0.54
cspg4	chondroitin sulfate proteoglycan 4	-2.224	-1.201	0.54
st6galnac3	ST6 (alpha-N-acetyl-neuraminyl-2,3-beta-galactosyl-1,3)-N-acetylgalactosaminide alpha-2,6-	-3.612	-1.952	0.54

	sialyltransferase 3			
col4a4	collagen, type IV, alpha 4	-4.166	-2.262	0.54
ccdc141	coiled-coil domain containing 141	-3.960	-2.152	0.54
tnc	tenascin C	-4.489	-2.440	0.54
rhobtb1	Rho-related BTB domain containing 1	-3.081	-1.679	0.54
lipa	lipase A, lysosomal acid, cholesterol esterase	-2.505	-1.367	0.55
setdb2	SET domain, bifurcated 2	-2.999	-1.637	0.55
sipa1l2	signal-induced proliferation-associated 1 like 2	-2.509	-1.377	0.55
kcnma1	potassium large conductance calcium-activated channel, subfamily M, alpha member 1	-2.723	-1.496	0.55
col14a1	collagen, type XIV, alpha 1	-2.995	-1.646	0.55
wipf1	WAS/WASL interacting protein family, member 1	-3.311	-1.826	0.55
ankrd28	ankyrin repeat domain 28	-2.874	-1.586	0.55
tgfb1i1	transforming growth factor beta 1 induced transcript 1	-3.733	-2.065	0.55
b3gnt5	UDP-GlcNAc:betaGal beta-1,3-N-acetylglucosaminyltransferase 5	-4.080	-2.259	0.55
cxadr	coxsackie virus and adenovirus receptor	-2.276	-1.262	0.55
metrnl	meteorin, glial cell differentiation regulator-like	-2.594	-1.440	0.56
LOC100909788	uncharacterized LOC100909788	-2.344	-1.303	0.56
lpar6	lysophosphatidic acid receptor 6	-3.551	-1.981	0.56
nrg1	neuregulin 1	-3.469	-1.943	0.56
rimklb	ribosomal modification protein rimK-like family member B	-3.498	-1.962	0.56
pcdh20	protocadherin 20	-3.644	-2.048	0.56
RGD1562284	glutaminyl-peptide cyclotransferase	-2.841	-1.598	0.56
dab2	Dab, mitogen-responsive phosphoprotein, homolog 2 (Drosophila)	-2.880	-1.625	0.56
zfp608	zinc finger protein 608	-4.445	-2.522	0.57
LOC361016	chromosome 3 open reading frame 67	-2.404	-1.364	0.57
RGD1309651	chromosome 16 open reading frame 74	-2.015	-1.145	0.57
gcom1	myocardial zonula adherens protein	-4.054	-2.310	0.57
col3a1	collagen, type III, alpha 1	-3.681	-2.099	0.57
lgals8	lectin, galactoside-binding, soluble, 8	-3.395	-1.940	0.57
esyt1	extended synaptotagmin-like protein 1	-2.804	-1.604	0.57
fads3	fatty acid desaturase 3	-3.330	-1.908	0.57
inadl	InaD-like (Drosophila)	-3.403	-1.952	0.57
tmem140	transmembrane protein 140	-2.960	-1.699	0.57
fabp3	fatty acid binding protein 3, muscle and heart (mammary-derived growth inhibitor)	-2.521	-1.448	0.57
hs6st1	heparan sulfate 6-O-sulfotransferase 1	-2.425	-1.401	0.58
tpm1	tropomyosin 1, alpha	-4.671	-2.700	0.58
akap5	A kinase (PRKA) anchor protein 5	-5.740	-3.319	0.58
faxdc2	fatty acid hydroxylase domain containing 2	-2.376	-1.375	0.58
ptprd	protein tyrosine phosphatase, receptor type, D	-2.275	-1.318	0.58
tubb2b	tubulin, beta 2B class IIb	-3.081	-1.787	0.58
itpr1	inositol 1,4,5-trisphosphate receptor, type 1	-2.330	-1.352	0.58
LOC100910319	uncharacterized LOC100910319	-4.831	-2.804	0.58
rasl11b	RAS-like, family 11, member B	-2.036	-1.182	0.58
ptprk	protein tyrosine phosphatase, receptor type, K	-2.245	-1.313	0.58
klhdc8b	kelch domain containing 8B	-2.798	-1.637	0.59
rbms3	RNA binding motif, single stranded interacting protein 3	-2.890	-1.691	0.59
six4	SIX homeobox 4	-4.106	-2.408	0.59
stc1	stanniocalcin 1	-6.415	-3.765	0.59
cd9	CD9 molecule	-2.155	-1.267	0.59
fgl2	fibrinogen-like 2	-2.920	-1.717	0.59
plxdc2	plexin domain containing 2	-2.909	-1.711	0.59
foxp2	forkhead box P2	-3.065	-1.805	0.59
arhgef25	Rho guanine nucleotide exchange factor (GEF) 25	-3.027	-1.784	0.59
pkp2	plakophilin 2	-2.840	-1.677	0.59
fam101b	family with sequence similarity 101, member B	-2.640	-1.561	0.59
slc24a3	solute carrier family 24 (sodium/potassium/calcium exchanger), member 3	-2.815	-1.669	0.59

slc31a2	solute carrier family 31 (copper transporter), member 2	-2.808	-1.669	0.59
LOC689770	C-type lectin domain family 2, member D	-3.265	-1.943	0.60
gpm6a	glycoprotein M6A	-2.061	-1.229	0.60
rell1	RELT-like 1	-2.236	-1.335	0.60
ppap2b	phosphatidic acid phosphatase type 2B	-3.764	-2.248	0.60
car3	carbonic anhydrase III, muscle specific	-10.956	-6.545	0.60
bmp4	bone morphogenetic protein 4	-5.959	-3.562	0.60
ppp2r1b	protein phosphatase 2, regulatory subunit A, beta	-2.440	-1.462	0.60
trim47	tripartite motif containing 47	-2.478	-1.490	0.60
fhl2	four and a half LIM domains 2	-3.512	-2.114	0.60
phex	phosphate regulating endopeptidase homolog, X-linked	-2.726	-1.642	0.60
s1pr2	sphingosine-1-phosphate receptor 2	-2.887	-1.741	0.60
LOC100911506	uncharacterized LOC100911506	-2.612	-1.578	0.60
arhgef26	Rho guanine nucleotide exchange factor (GEF) 26	-3.018	-1.825	0.60
spink8	serine peptidase inhibitor, Kazal type 8 (putative)	-11.814	-7.174	0.61
pam	peptidylglycine alpha-amidating monooxygenase	-2.200	-1.339	0.61
reln	reelin	-2.272	-1.383	0.61
cebpd	CCAAT/enhancer binding protein (C/EBP), delta	-4.080	-2.486	0.61
slc2a13	solute carrier family 2 (facilitated glucose transporter), member 13	-2.445	-1.497	0.61
cobl	cordon-bleu WH2 repeat protein	-2.554	-1.565	0.61
jdp2	Jun dimerization protein 2	-2.151	-1.326	0.62
ddah1	dimethylarginine dimethylaminohydrolase 1	-2.323	-1.435	0.62
tmeff1	transmembrane protein with EGF-like and two follistatin-like domains 1	-3.238	-2.004	0.62
sh3bp4	SH3-domain binding protein 4	-2.541	-1.574	0.62
LOC100912462	uncharacterized LOC100912462	-3.001	-1.859	0.62
amotl2	angiomotin like 2	-2.691	-1.668	0.62
pip4k2a	phosphatidylinositol-5-phosphate 4-kinase, type II, alpha	-2.657	-1.647	0.62
cpq	carboxypeptidase Q	-2.753	-1.707	0.62
scn1a	sodium channel, voltage-gated, type I, alpha subunit	-2.508	-1.556	0.62
rhoj	ras homolog family member J	-2.102	-1.307	0.62
ramp1	receptor (G protein-coupled) activity modifying protein 1	-2.386	-1.484	0.62
LOC684173	TNF receptor-associated factor 5	-3.430	-2.137	0.62
gca	grancalcin, EF-hand calcium binding protein	-2.857	-1.782	0.62
cd200	CD200 molecule	-2.867	-1.791	0.62
LOC100912373	uncharacterized LOC100912373	-3.338	-2.096	0.63
tmcc3	transmembrane and coiled-coil domain family 3	-3.187	-2.004	0.63
cst3	cystatin C	-2.604	-1.638	0.63
bche	butyrylcholinesterase	-2.847	-1.793	0.63
maff	v-maf avian musculoaponeurotic fibrosarcoma oncogene homolog F	-2.823	-1.780	0.63
stxbp6	syntaxin binding protein 6 (amisyn)	-4.123	-2.604	0.63
nupr1	nuclear protein, transcriptional regulator, 1	-2.145	-1.356	0.63
col4a5	collagen, type IV, alpha 5	-2.521	-1.594	0.63
ppic	peptidylprolyl isomerase C (cyclophilin C)	-3.238	-2.057	0.64
sowahc	sosondowah ankyrin repeat domain family member C	-2.419	-1.537	0.64
ptp4a3	protein tyrosine phosphatase type IVA, member 3	-3.758	-2.400	0.64
LOC100912470	uncharacterized LOC100912470	-2.405	-1.538	0.64
LOC684871	chromosome 8 open reading frame 4	-2.231	-1.428	0.64
wwc1	WW and C2 domain containing 1	-2.320	-1.485	0.64
rgs7bp	regulator of G-protein signaling 7 binding protein	-5.807	-3.719	0.64
hspb1	heat shock 27kDa protein 1	-3.293	-2.110	0.64
slc8a1	solute carrier family 8 (sodium/calcium exchanger), member 1	-3.022	-1.939	0.64
serpinb6b	serine (or cysteine) peptidase inhibitor, clade B, member 6b	-3.048	-1.958	0.64
gls	glutaminase	-2.559	-1.644	0.64
zfpm2	zinc finger protein, FOG family member 2	-2.345	-1.508	0.64
bmp2	bone morphogenetic protein 2	-2.051	-1.320	0.64
sclt1	sodium channel and clathrin linker 1	-2.625	-1.691	0.64

csrp1	cysteine and glycine-rich protein 1	-3.724	-2.399	0.64
serpinb6	serpin peptidase inhibitor, clade B (ovalbumin), member 6	-2.873	-1.852	0.64
mal2	mal, T-cell differentiation protein 2 (gene/pseudogene)	-3.556	-2.293	0.64
fam159b	family with sequence similarity 159, member B	-2.276	-1.472	0.65
etl4	KIAA1217	-2.751	-1.780	0.65
itga6	integrin, alpha 6	-2.115	-1.369	0.65
cttnbp2	cortactin binding protein 2	-2.067	-1.340	0.65
phlpp1	PH domain and leucine rich repeat protein phosphatase 1	-2.216	-1.440	0.65
meis1	Meis homeobox 1	-2.277	-1.484	0.65
pkig	protein kinase (cAMP-dependent, catalytic) inhibitor gamma	-2.433	-1.586	0.65
rab11fip1	RAB11 family interacting protein 1 (class I)	-2.568	-1.676	0.65
dsp	desmoplakin	-2.749	-1.795	0.65
fam124a	family with sequence similarity 124A	-2.392	-1.562	0.65
hdac5	histone deacetylase 5	-3.595	-2.354	0.65
angpt1	angiopoietin 1	-4.010	-2.629	0.66
hbegf	heparin-binding EGF-like growth factor	-6.384	-4.191	0.66
slc43a1	solute carrier family 43 (amino acid system L transporter), member 1	-2.213	-1.456	0.66
mitf	microphthalmia-associated transcription factor	-3.143	-2.069	0.66
akt3	v-akt murine thymoma viral oncogene homolog 3	-2.311	-1.525	0.66
gadd45b	growth arrest and DNA-damage-inducible, beta	-5.789	-3.826	0.66
foxo1	forkhead box O1	-2.133	-1.412	0.66
ank2	ankyrin 2, neuronal	-2.895	-1.922	0.66
il15	interleukin 15	-2.082	-1.383	0.66
samd11	sterile alpha motif domain containing 11	-2.071	-1.377	0.66
tslp	thymic stromal lymphopoietin	-3.707	-2.468	0.67
tbc1d23	TBC1 domain family, member 23	-2.902	-1.934	0.67
tspan2	tetraspanin 2	-4.651	-3.107	0.67
cnrip1	cannabinoid receptor interacting protein 1	-2.140	-1.432	0.67
fam20c	family with sequence similarity 20, member C	-2.052	-1.377	0.67
perp	PERP, TP53 apoptosis effector	-2.421	-1.628	0.67
col5a2	collagen, type V, alpha 2	-2.490	-1.675	0.67
b3galnt1	beta-1,3-N-acetylgalactosaminyltransferase 1 (globoside blood group)	-2.625	-1.766	0.67
pcbd1	pterin-4 alpha-carbinolamine dehydratase/dimerization cofactor of hepatocyte nuclear factor 1 alpha	-2.211	-1.488	0.67
ppp1r1a	protein phosphatase 1, regulatory (inhibitor) subunit 1A	-2.185	-1.472	0.67
klf6	Kruppel-like factor 6	-3.565	-2.403	0.67
irs1	insulin receptor substrate 1	-2.468	-1.668	0.68
rnf180	ring finger protein 180	-2.599	-1.763	0.68
magee1	melanoma antigen family E, 1	-2.265	-1.537	0.68
actn1	actinin, alpha 1	-3.247	-2.208	0.68
mical1	microtubule associated monooxygenase, calponin and LIM domain containing 1	-2.866	-1.958	0.68
eif4e3	eukaryotic translation initiation factor 4E family member 3	-2.980	-2.037	0.68
dock5	dedicator of cytokinesis 5	-2.867	-1.971	0.69
foxp1	forkhead box P1	-2.812	-1.935	0.69
arx	aristaless related homeobox	-3.520	-2.423	0.69
tmem136	transmembrane protein 136	-2.036	-1.403	0.69
dock11	dedicator of cytokinesis 11	-2.860	-1.971	0.69
ccdc80	coiled-coil domain containing 80	-2.519	-1.736	0.69
flnb	filamin B, beta	-2.413	-1.664	0.69
pea15	phosphoprotein enriched in astrocytes 15	-2.064	-1.425	0.69
morc4	MORC family CW-type zinc finger 4	-2.312	-1.598	0.69
mterfd3	MTERF domain containing 3	-2.400	-1.664	0.69
ajuba	ajuba LIM protein	-2.180	-1.513	0.69
rab30	RAB30, member RAS oncogene family	-2.984	-2.075	0.70
tcf7l2	transcription factor 7-like 2 (T-cell specific, HMG-box)	-2.176	-1.515	0.70
cpne8	copine VIII	-2.411	-1.682	0.70

cntrl	centriolin	-2.136	-1.491	0.70
svil	supervillin	-2.048	-1.431	0.70
spats2l	spermatogenesis associated, serine-rich 2-like	-3.854	-2.694	0.70
LOC100909928	uncharacterized LOC100909928	-2.322	-1.625	0.70
tmem98	transmembrane protein 98	-2.096	-1.467	0.70
fstl1	follistatin-like 1	-2.146	-1.503	0.70
nexn	nexilin (F actin binding protein)	-6.872	-4.824	0.70
cpe	carboxypeptidase E	-4.147	-2.922	0.70
RGD1559896	chromosome 1 open reading frame 198	-2.995	-2.111	0.70
pon3	paraoxonase 3	-2.074	-1.462	0.70
rin2	Ras and Rab interactor 2	-2.413	-1.701	0.70
emp2	epithelial membrane protein 2	-2.110	-1.488	0.71
vcl	vinculin	-2.635	-1.859	0.71
synj2	synaptojanin 2	-2.337	-1.649	0.71
anxa1	annexin A1	-2.139	-1.514	0.71
zfp36l2	ZFP36 ring finger protein-like 2	-2.511	-1.778	0.71
pdlim1	PDZ and LIM domain 1	-2.507	-1.776	0.71
celf2	CUGBP, Elav-like family member 2	-3.195	-2.264	0.71
ankrd44	ankyrin repeat domain 44	-2.014	-1.430	0.71
apbb1	amyloid beta (A4) precursor protein-binding, family B, member 1 (Fe65)	-2.063	-1.465	0.71
nacad	NAC alpha domain containing	-3.534	-2.511	0.71
gnai1	guanine nucleotide binding protein (G protein), alpha inhibiting activity polypeptide 1	-2.180	-1.549	0.71
myadm	myeloid-associated differentiation marker	-2.974	-2.122	0.71
alcam	activated leukocyte cell adhesion molecule	-2.833	-2.030	0.72
ndrg2	NDRG family member 2	-2.172	-1.557	0.72
pycard	PYD and CARD domain containing	-2.019	-1.450	0.72
ptpla	protein tyrosine phosphatase-like (proline instead of catalytic arginine), member A	-3.638	-2.616	0.72
myo1c	myosin IC	-2.994	-2.156	0.72
c1qtnf7	C1q and tumor necrosis factor related protein 7	-4.259	-3.070	0.72
tdrp	testis development related protein	-2.006	-1.446	0.72
ndrg4	NDRG family member 4	-2.021	-1.457	0.72
rasgrp3	RAS guanyl releasing protein 3 (calcium and DAG-regulated)	-6.037	-4.354	0.72
LOC100910189	uncharacterized LOC100910189	-2.753	-1.987	0.72
ripply1	ripply1 homolog (zebrafish)	-2.836	-2.048	0.72
LOC100361913	RIKEN cDNA 2200002D01 gene	-2.562	-1.852	0.72
cldn2	claudin 2	-5.555	-4.027	0.72
Phf17	jade family PHD finger 1	-2.224	-1.608	0.72
LOC100912219	uncharacterized LOC100912219	-2.603	-1.888	0.73
tmem106a	transmembrane protein 106A	-2.283	-1.659	0.73
stard13	StAR-related lipid transfer (START) domain containing 13	-8.422	-6.124	0.73
slc12a2	solute carrier family 12 (sodium/potassium/chloride transporter), member 2	-2.360	-1.717	0.73
hectd2	HECT domain containing E3 ubiquitin protein ligase 2	-2.590	-1.885	0.73
nipal2	NIPA-like domain containing 2	-2.049	-1.493	0.73
stx3	syntaxin 3	-2.703	-1.972	0.73
lima1	LIM domain and actin binding 1	-2.535	-1.850	0.73
fez2	fasciculation and elongation protein zeta 2 (zygin II)	-2.021	-1.477	0.73
dzip1l	DAZ interacting zinc finger protein 1-like	-2.296	-1.678	0.73
zc2hc1a	zinc finger, C2HC-type containing 1A	-2.995	-2.190	0.73
zfp445	zinc finger protein 445	-2.007	-1.468	0.73
prnp	prion protein	-2.253	-1.653	0.73
cnst	consortin, connexin sorting protein	-2.590	-1.904	0.74
col8a1	collagen, type VIII, alpha 1	-2.338	-1.719	0.74
sun2	Sad1 and UNC84 domain containing 2	-2.192	-1.616	0.74
thbd	thrombomodulin	-2.925	-2.162	0.74
plcd1	phospholipase C, delta 1	-2.037	-1.521	0.75
itgb3	integrin, beta 3 (platelet glycoprotein IIIa, antigen CD61)	-2.390	-1.785	0.75

rnd3	Rho family GTPase 3	-2.351	-1.759	0.75
chn1	chimerin 1	-2.507	-1.879	0.75
gypc	glycophorin C (Gerbich blood group)	-2.492	-1.873	0.75
pkia	protein kinase (cAMP-dependent, catalytic) inhibitor alpha	-3.027	-2.279	0.75
gnb4	guanine nucleotide binding protein (G protein), beta polypeptide 4	-2.356	-1.775	0.75
basp1	brain abundant, membrane attached signal protein 1	-2.068	-1.560	0.75
myl4	myosin, light chain 4, alkali; atrial, embryonic	-2.056	-1.560	0.76
zyx	zyxin	-2.309	-1.756	0.76
LOC100362894	hypothetical protein LOC100362894	-2.465	-1.878	0.76
anxa3	annexin A3	-3.018	-2.300	0.76
ahnak	AHNAK nucleoprotein	-2.083	-1.590	0.76
fxyd6	FXYD domain containing ion transport regulator 6	-2.091	-1.601	0.77
atp10a	ATPase, class V, type 10A	-2.298	-1.761	0.77
nap1l3	nucleosome assembly protein 1-like 3	-2.049	-1.572	0.77
csgalnact2	chondroitin sulfate N-acetylgalactosaminyltransferase 2	-2.333	-1.794	0.77
farp1	FERM, RhoGEF (ARHGEF) and pleckstrin domain protein 1 (chondrocyte-derived)	-2.046	-1.576	0.77
LOC100910396	uncharacterized LOC100910396	-2.162	-1.669	0.77
pls3	plastin 3	-2.134	-1.652	0.77
map1b	microtubule-associated protein 1B	-2.042	-1.584	0.78
acta2	actin, alpha 2, smooth muscle, aorta	-3.181	-2.473	0.78
slc38a4	solute carrier family 38, member 4	-3.148	-2.456	0.78
hip1	huntingtin interacting protein 1	-2.723	-2.126	0.78
tes	testis derived transcript (3 LIM domains)	-2.498	-1.961	0.79
vldlr	very low density lipoprotein receptor	-2.231	-1.752	0.79
cr1l	complement component (3b/4b) receptor 1-like	-2.021	-1.589	0.79
rabgap1l	RAB GTPase activating protein 1-like	-2.150	-1.691	0.79
clic4	chloride intracellular channel 4	-2.310	-1.817	0.79
edn1	endothelin 1	-6.288	-4.948	0.79
pxdc1	PX domain containing 1	-2.425	-1.914	0.79
kank2	KN motif and ankyrin repeat domains 2	-2.078	-1.643	0.79
fgf13	fibroblast growth factor 13	-2.085	-1.650	0.79
rras2	related RAS viral (r-ras) oncogene homolog 2	-2.138	-1.692	0.79
nek6	NIMA-related kinase 6	-2.236	-1.770	0.79
myh9	myosin, heavy chain 9, non-muscle	-2.218	-1.756	0.79
ptger2	prostaglandin E receptor 2 (subtype EP2), 53kDa	-3.654	-2.900	0.79
LOC500300	chromosome 10 open reading frame 10	-3.555	-2.823	0.79
ankh	ANKH inorganic pyrophosphate transport regulator	-2.002	-1.594	0.80
dpysl3	dihydropyrimidinase-like 3	-4.249	-3.396	0.80
ldb3	LIM domain binding 3	-2.583	-2.067	0.80
sema3a	sema domain, immunoglobulin domain (Ig), short basic domain, secreted, (semaphorin) 3A	-2.144	-1.717	0.80
rrad	Ras-related associated with diabetes	-2.250	-1.802	0.80
atf3	activating transcription factor 3	-2.141	-1.717	0.80
irs2	insulin receptor substrate 2	-2.415	-1.938	0.80
tmem51	transmembrane protein 51	-2.008	-1.613	0.80
ankrd1	ankyrin repeat domain 1 (cardiac muscle)	-4.748	-3.827	0.81
LOC100362819	autism susceptibility candidate 2	-2.380	-1.920	0.81
chmp4c	charged multivesicular body protein 4C	-2.405	-1.942	0.81
rnd1	Rho family GTPase 1	-2.513	-2.031	0.81
gata3	GATA binding protein 3	-3.144	-2.543	0.81
pdgfc	platelet derived growth factor C	-2.267	-1.838	0.81
pdlim7	PDZ and LIM domain 7 (enigma)	-2.210	-1.796	0.81
mef2c	myocyte enhancer factor 2C	-3.346	-2.728	0.82
gpnmb	glycoprotein (transmembrane) nmb	-2.083	-1.699	0.82
gpr176	G protein-coupled receptor 176	-2.250	-1.841	0.82
spry1	sprouty homolog 1, antagonist of FGF signaling (Drosophila)	-2.366	-1.941	0.82
MGC95152	similar to B230212L03Rik protein	-2.180	-1.779	0.82

actn4	actinin, alpha 4	-2.472	-2.040	0.83
actg2	actin, gamma 2, smooth muscle, enteric	-2.757	-2.291	0.83
sertad4	SERTA domain containing 4	-2.545	-2.116	0.83
rasa1	RAS p21 protein activator (GTPase activating protein) 1	-2.072	-1.728	0.83
s1pr1	sphingosine-1-phosphate receptor 1	-8.059	-6.742	0.84
plcxd2	phosphatidylinositol-specific phospholipase C, X domain containing 2	-2.179	-1.840	0.84
scn9a	sodium channel, voltage-gated, type IX, alpha subunit	-4.290	-3.628	0.85
f2r	coagulation factor II (thrombin) receptor	-2.096	-1.773	0.85
slc40a1	solute carrier family 40 (iron-regulated transporter), member 1	-2.037	-1.724	0.85
lxn	latexin	-2.436	-2.067	0.85
lphn3	latrophilin 3	-2.606	-2.213	0.85
tead3	TEA domain family member 3	-2.056	-1.749	0.85
lpar4	lysophosphatidic acid receptor 4	-3.060	-2.612	0.85
trpc4	transient receptor potential cation channel, subfamily C, member 4	-2.029	-1.732	0.85
arid5b	AT rich interactive domain 5B (MRF1-like)	-3.669	-3.157	0.86
map3k8	mitogen-activated protein kinase kinase kinase 8	-2.002	-1.731	0.86
camta1	calmodulin binding transcription activator 1	-2.572	-2.247	0.87
kctd9	potassium channel tetramerization domain containing 9	-2.068	-1.810	0.88
sbspon	somatomedin B and thrombospondin, type 1 domain containing	-2.893	-2.540	0.88
pla2r1	phospholipase A2 receptor 1, 180kDa	-2.085	-1.834	0.88
bbs7	Bardet-Biedl syndrome 7	-2.022	-1.789	0.88
plcxd3	phosphatidylinositol-specific phospholipase C, X domain containing 3	-2.061	-1.825	0.89
cnksr3	CNKSR family member 3	-3.084	-2.733	0.89
rassf3	Ras association (RalGDS/AF-6) domain family member 3	-2.112	-1.885	0.89
pcgf5	polycomb group ring finger 5	-2.781	-2.487	0.89
scrn1	secernin 1	-2.120	-1.897	0.89
cryab	crystallin, alpha B	-2.500	-2.242	0.90
car5b	carbonic anhydrase VB, mitochondrial	-2.050	-1.843	0.90
rock2	Rho-associated, coiled-coil containing protein kinase 2	-2.227	-2.009	0.90
utrn	utrophin	-2.037	-1.861	0.91
scn7a	sodium channel, voltage-gated, type VII, alpha subunit	-2.503	-2.289	0.91
acta1	actin, alpha 1, skeletal muscle	-5.001	-4.596	0.92
myl9	myosin, light chain 9, regulatory	-2.159	-1.987	0.92
lrch1	leucine-rich repeats and calponin homology (CH) domain containing 1	-3.168	-2.938	0.93
adamts12	ADAM metallopeptidase with thrombospondin type 1 motif, 12	-3.278	-3.084	0.94
glipr1	GLI pathogenesis-related 1	-2.012	-1.921	0.95
vtcn1	V-set domain containing T cell activation inhibitor 1	-2.387	-2.309	0.97
f3	coagulation factor III (thromboplastin, tissue factor)	-3.560	-3.476	0.98
abcd2	ATP-binding cassette, sub-family D (ALD), member 2	-2.139	-2.100	0.98
klf7	Kruppel-like factor 7 (ubiquitous)	-2.234	-2.257	1.01
tnnc1	troponin C type 1 (slow)	-3.005	-3.063	1.02
testin	RIKEN cDNA 4930486L24 gene	-2.778	-2.875	1.03
Ghr	Growth Hormone receptor	-1.932	-2.067	1.07
trpc3	transient receptor potential cation channel, subfamily C, member 3	-2.473	-2.656	1.07
Cnn1	Calponin 1	-1.920	-2.089	1.09
tjp2	tight junction protein 2	-2.054	-2.268	1.10
tpm2	tropomyosin 2, beta	-2.321	-2.673	1.15
Hipk3	Homeodomain Interacting Protein Kinase 3	-1.864	-2.221	1.19
Arg1	Arginase 1	-1.881	-2.285	1.21
Gadd45g	Growth Aresst and DNA-Damage Inducible	-1.567	-2.039	1.30
tns3	tensin 3	-2.035	-2.700	1.33
lrrc39	leucine rich repeat containing 39	-3.304	-4.767	1.44
Fam198b	family with sequence similarity 198, member B	-1.396	-2.341	1.68
RGD1305254	cell migration inducing protein, hyaluronan binding	-2.127	-3.616	1.70

Supplementary Table S2 Major functional categories and the associated genes identified by DAVID analysis of transcripts regulated to a similar extent by FSH stimulation or PKA activation

**Functional Categories**	**Gene Symbol**
Cholesterol biosynthesis	CYP51, EBP, MVD, DHCR7, HMGCS1, MVK, CFTR, LSS, IDI1, HSD17B7, FDFT1
Steroid biosynthesis	CYP51, EBP, MVD, HSD17B1, DHCR7, HMGCS1, MVK, LSS, IDI1, HSD17B7, FDFT1
Actin cytoskeleton	MICALL2, LIMA1, ACTA1, PDLIM7, ACTN4, CRYAB, ACTN1, ZYX, MYH9, FLNB, VCL, CDC42EP2, TPM1, RASA1
Apoptosis	MEF2C, GCLC, MITF, FOXO1, CX3CL1, TIMP3, GCH1, EDNRB, ALDH1A3, PYCARD, PCSK9, NQO1, DDAH1, RASA1, IHH, GHR, HIP1, EGFR, BMP4, KCNMA1, PGAP2, HERPUD1, SGK1, CEBPB, ACTN4, MADD, CRYAB, MSH2, SPHK1, ANXA1, ITGA1, CST3, NR4A1, INHA, GAL, SOD2, INHBA, PLA2G4A, NOTCH1, NUPR1, F3, HIPK3, HSPB1, HSPD1, PERP, PRNP, APBB1, F2R
Regulation of Transcription	MEF2C, THRB, MITF, FOXO1, NPAS2, S1PR1, GATA3, GATA4, RUNX1, DDAH1, IHH, NR1H3, BMP4, SREBF1, KLF6, KLF7, BMP2, CEBPB, CEBPD, PCBD1, MAFB, NR4A1, SIX4, TEAD3, INHBA, NOTCH1, ZFPM2, NR5A2, APBB1, F2R, NR5A1
Protein kinases	EGFR, IRAK2, SGK1, ROCK2, MAPKAPK3, EPHB3, IRS1, PRKCB, RPS6KA5, HUNK, PLK2, SBK1, HIPK3, MAP3K8, RELN, DYRK3, GRK5, NEK6, DCLK1, AKT3
Chromatin organization,	HDAC5, RPS6KA5, PHF17, HOPX, NAP1L3, APBB1
G1/S transition of mitotic cell cycle	CCND2, ID4, EGFR, INHBA,

Supplementary Table S3. Major functional categories and the associated genes identified by DAVID analysis of transcripts that are regulated more effectively by FSH stimulation than PKA-CQR expression.

**Functional Categories**	**Gene Symbol**
Ion Homeostasis	AGTR2, CAV1, PTGER3, CCL2, ADM, AGTR1A, RYR2, LPAR1
Extracellular matrix binding	NID1, DCN, SPP1, CYR61
Apoptosis	AGTR2, BDNF, CCL2, HTRA2, ETS1, ARNT2, CLU, CIDEA, CD74, TGFB2, FN1
Cytoskeleton Organization	CAV1, SORBS1, CALD1, PDLIM3, GAS7
Regulation of Transcription	NFE2, MEOX2, ETS1, ARNT2, LRRFIP1, PAWR, NR0B1

Supplementary Table S4. Major functional categories and the associated genes identified by DAVID analysis of transcripts that are regulated more effectively by PKA-CQR expression than by by FSH stimulation.

**Functional Categories**	**Gene Symbol**
Ovulation	AREG, EREG, PGR, SNAP25,TNFAIP6, PTGS2
Transition metal Binding	NR4A3, PGR, CYP19A1, PTGS2, SCD
Integral to membrane	AREG, GJA5, HAVCR1, NPY1R, SLC7a11, SCD

Supplementary table S5: List of antibodies and conditions used for immunoblotting.

ANTIBODY	Supplier P/N	BLOCK & Conditions	1° ANTIBODY Dilution & Conditions	Post 1° WASHES	2°ANTIBODY Dilution & Conditions	Post 2° WASHES
**Akt (PKB)**	Cell Signaling #9272	0.5% Block/TBST<br>1-2hr RT	1:1000 in 5% BSA/TBST<br>O/N 4°C	TBST 3x 5min	SIGMA **Anti-Rabbit** IgG-POD<br>1:10,000 in 0.5% Block/TBST<br>1.5hr RT	TBST 3x 5min
**P-Akt (Ser473)**	Cell Signaling #9271	0.5% Block/TBST<br>1-2hr RT	1:500 in 5% BSA/TBST<br>O/N 4°C	TBST 3x 5min	SIGMA **Anti-Rabbit** IgG-POD<br>1:10,000 in 0.5% Block/TBST<br>1.5hr RT	TBST 3x 5min
**P-Akt (Thr308)**	Cell Signaling #9275	5% Milk/TBS<br>1-2hr RT	1:500 in 5% Milk/TBST + 0.1% Tween + azi	TBS 3x 10min	**Anti-Rabbit** IgG-POD<br>1:500 in 5% Milk/TBS	TBS 3x 10min
**β-Catenin**	Santa Cruz #7199	3% BSA/TBST<br>1-2hr RT	1:1000 in 3% BSA/TBST<br>O/N 4°C	TBST 3x 5min	SIGMA **Anti-Rabbit** IgG-POD<br>1:10,000 in 3% BSA/TBST<br>1.5hr RT	TBST 3x 5min
**P-β-Catenin (Ser552)**	Cell Signaling #9566	3% BSA/TBST<br>1-2hr RT	1:1000 in 3% BSA/TBST<br>O/N 4°C	TBST 3x 5min	SIGMA **Anti-Rabbit** IgG-POD<br>1:10,000 in 3% BSA/TBST<br>1.5hr RT	TBST 3x 5min
**CREB**	Millipore #06-863	3% Milk/PBS<br>1-2hr RT	1:1000 in 3% Milk/PBS<br>O/N 4°C	MQ-H_2O 2x 5min	SIGMA **Anti-Rabbit** IgG-POD<br>1:10,000 in 3% Milk/PBS<br>1.5hr RT	MQ-H_2O 2x 5min<br>0.05% Tween/PBS 1x 5min<br>MQ-H_2O 5x Rinse
**P-CREB (Ser133)**	Millipore #05-667	3% Milk/TBS<br>1-2hr RT	1:100 in 3% Milk/TBS<br>O/N 4°C	MQ-H_2O 2x 5min	SIGMA **Anti-Mouse** IgG-POD<br>1:10,000 in 3% Milk/TBS<br>1.5hr RT	MQ-H_2O 2x 5min<br>0.05% Tween/TBS 1x 5min<br>MQ-H_2O 5x Rinse
**FoxO1**	Cell Signaling #2880	5% Milk/TBST<br>1-2hr RT	1:2000 in 5% BSA/TBST<br>O/N 4°C	TBST 3x 5min	SIGMA **Anti-Rabbit** IgG-POD<br>1:10,000 in 5% Milk/TBST<br>1.5hr RT	TBST 3x 5min
**P-FoxO1 (Ser256)**	Santa Cruz #sc101681	0.5% Block/TBST<br>1-2hr RT	1:100 in 5% BSA/TBST<br>O/N 4°C	TBST 3x 5min	SIGMA **Anti-Rabbit** IgG-POD<br>1:10,000 in 0.5% Block/TBST<br>1.5hr RT	TBST 3x 5min
**GAB2**	EMD Millipore #06-967	5% Milk/TBS<br>1-2 hr RT	1:1000 in 5% Milk/TBS + azide	TBS 3x 10min	**Anti-Rabbit** IgG-POD<br>1:1500 in 5% Milk/TBS	TBS 3x 10min
**P-GAB2 (Ser159)**	Cell Signaling #3884	5% Milk/TBS<br>1-2 hr RT	1:1000 in 5% Milk/TBS + azide	TBS 3x 10min	**Anti-Rabbit** IgG-POD<br>1:1000 in 5% Milk/TBS	TBS 3x 10min
**GSK-3β**	Cell Signaling #9315	5% Milk/TBS<br>1-2 hr RT	1:1000 in 5% Milk/TBS + azide	TBS 3x 10min	**Anti-Rabbit** IgG-POD<br>1:1000 in 5% Milk/TBS	TBS 3x 10min
**P-GSK-3β (Ser9)**	Cell Signaling #9336	5% Milk/TBS<br>1-2 hr RT	1:1000 in 5% Milk/TBS + azide	TBS 3x 10min	**Anti-Rabbit** IgG-POD<br>1:1000 in 5% Milk/TBS	TBS 3x 10min
**P-p38 (Thr180/Tyr182)<br>(MAPK)**	Cell Signaling #4511<br>(Rab mAb)	5% Milk/TBST<br>1-2hr RT	1:1000 in 5% BSA/TBST<br>O/N 4°C	TBST 3x 5min	SIGMA **Anti-Rabbit** IgG-POD<br>1:10,000 in 5% Milk/TBST<br>1.5hr RT	TBST 3x 5min
**p42/44<br>(ERK 1&2)**	Millipore #06-182	3% Milk/PBS<br>1-2hr RT	1:10,000 in 3% Milk/PBS<br>O/N 4°C	MQ-H_2O 2x 5min	SIGMA **Anti-Rabbit** IgG-POD<br>1:10,000 in 3% Milk/PBS<br>1.5hr RT	MQ-H_2O 2x 5min<br>0.05% Tween/PBS 1x 5min<br>MQ-H_2O 5x Rinse
**P-p42/44 (Thr202/Tyr204)<br>(P-ERK 1&2)**	Cell Signaling #9109	0.5% Block/TBST<br>1-2hr RT	1:2000 in 5% BSA/TBST<br>O/N 4°C	TBST 3x 5min	SIGMA **Anti-Rabbit** IgG-POD<br>1:10,000 in 0.5% Block/TBST<br>1.5hr RT	TBST 3x 5min
**PKAc**	Transduction Labs #610981<br>(BD)	0.5% Block/TBS<br>1-2hr RT	1:500 in 0.5% Block/TBS<br>O/N 4°C	TBST 3x 5min	SIGMA **Anti-Mouse** IgG-POD<br>1:10,000 in 0.5% Block/TBS<br>1.5hr RT	TBST 3x 5min
**cSrc**	Santa Cruz #sc18	3% Milk/TBST<br>1-2hr RT	1:1000 in 3% Milk/TBST<br>O/N 4°C	TBST 3x 5min	SIGMA **Anti-Rabbit** IgG-POD<br>1:10,000 in 3% Milk/TBST<br>1.5hr RT	TBST 3x 5min
**P-Src (Tyr416)**	Cell Signaling #6943	5% Milk/TBST<br>1-2hr RT	1:1000 in 5% BSA/TBST<br>O/N 4°C	TBST 3x 5min	SIGMA **Anti-Rabbit** IgG-POD<br>1:10,000 in 5% Milk/TBST<br>1.5hr RT	TBST 3x 5min
**YAP**	Cell Signaling #12395	3% BSA/TBST<br>1-2hr RT	1:1000 in 3% BSA/TBST<br>O/N 4°C	TBST 3x 5min	SIGMA **Anti-Mouse** IgG-POD<br>1:10,000 in 3% BSA/TBST<br>1.5hr RT	TBST 3x 5min
**P-YAP (Ser127)**	Cell Signaling #13008	3% BSA/TBST<br>1-2hr RT	1:1000 in 3% BSA/TBST<br>O/N 4°C	TBST 3x 5min	SIGMA **Anti-Rabbit** IgG-POD<br>1:10,000 in 3% BSA/TBST<br>1.5hr RT	TBST 3x 5min
